# Developing China’s Ecological Redline Policy using ecosystem services assessments for land use planning

**DOI:** 10.1038/s41467-018-05306-1

**Published:** 2018-08-02

**Authors:** Yang Bai, Christina P. Wong, Bo Jiang, Alice C. Hughes, Min Wang, Qing Wang

**Affiliations:** 1 Center for Integrative Conservation, Xishuangbanna Tropical Botanical Garden, Chinese Academy of Sciences, Menglun, 666303 China; 2 State Key Laboratory of Urban and Regional Ecology, Research Center for Eco-Environmental Sciences, Chinese Academy of Sciences, Beijing, 100085 China; 3 Changjiang Water Resources Protection Institute, Wuhan, 430051 China; 4 Institute of Applied Ecology, Shanghai Academy of Environmental Sciences, Shanghai, 200233 China

## Abstract

Ecosystems services (ES) assessment is a significant scientific topic recognized for its potential to address sustainability issues. However, there is an absence of science–policy frameworks in land use planning that lead to the ES science being used in policy. China’s Ecological Redline Policy (ERP) is one of the first national policies utilizing multiple ES, but there is no standardized approach for working across the science–policy interface. We propose a transdisciplinary framework to determine ecological redline areas (ERAs) in Shanghai using: ES, biodiversity and ecologically fragile hotspots, landscape structure, and stakeholder opinions. We determine the five criteria to identify ERAs for Shanghai using multi-temporal, high resolution images (0.5 m) and biophysical models. We examine ERP effectiveness by comparing land use scenarios for 2040. Compared to alternative land uses, ES increase significantly under the ERP. The inclusion of ES in spatial planning led stakeholders to increase terrestrial habitat protection by 174% in Shanghai. Our analysis suggests that strategic planning for ES could reduce tradeoffs between environmental quality and development.

## Abstract (web summary)

Ecosystem services, though a prominent ecological concept, have yet to be considered in major land use policy changes. Here, the authors demonstrate how the Ecological Redline Policy implemented in Shanghai utilized ecosystem services science and stakeholder engagement.

## Introduction

Ecosystem services (ES) assessments offer a means of practicing integrated approaches to address the serious policy challenge of incorporating environmental issues into development decisions^1,2^. To promote wise decision-making, policymakers need information from scientists about how different land use decisions may affect the condition of ecosystems and the flow of ES^3^. A major challenge is developing capacities on ES assessments for spatial planning, which is difficult because ES requires new interdisciplinary methodologies to assess multiple environmental and social concerns^4^.

Despite increasing political interest in ES, the use of ES information remains quite limited^5–8^. National ES assessments have been conducted in China^9^, United Kingdom^10^, and Mexico^11^. Scientists have conducted regional ES assessments for spatial planning in Pampas (Argentina)^12^, Hawaii (USA)^13^, Boredeux (France)^14^, Vancouver Island (Canada)^15^, Tampere (Finland)^16^, and coastal areas in Belize^17^. A number of these studies use the Integrated Valuation of Ecosystem Services and Tradeoffs models (InVEST) to assess ES, but few demonstrate an application by decision-makers for determiningland use planning targets^8,18^. Albert et al.^6^ state the research priority is developing transdisciplinary case studies of application to advance ES tools and methods for real-world planning.

One institutional obstacle is the lack of ES standards (i.e., assessment protocols and targets)^19^. Many researchers suggest scientists focus their work on municipalities to develop the required case studies for ES standards since cities are critical administrative units for land use planning^6,20,21^. However Hansen et al.^20^ show ES assessments are only being used as a supporting concept in urban planning. There are no published examples of a municipality implementing spatial plans based on ES assessment. However, a unique opportunity is emerging in China where municipalities want ES information to meet sustainability goals. China is the first major economy to formulate a national policy, mandating governments to establish ES assessments in land use planning known as the Ecological Redline Policy (ERP)^22,23^.

China is experiencing an environmental crisis, and President Xi realizes China must transform its development approach from “grow first, clean up later” to the “ecological civilization” where development respects ecological carrying capacities^24,25^. ERP seeks to sustain critical ES for social welfare using coordinated planning at a national scale. The policy instrument is establishing key ecological function zones (EFZs) using ecological redline areas (ERAs)^26^. EFZs were selected to sustain five national ES: biodiversity conservation, water resources conservation, flood mitigation, soil conservation, and sandstorm prevention^27^. ERAs represent an attempt at establishing ES assessment standards in land use planning, defined as the “minimum ecological area needed to guarantee and maintain ecological safety and functionality, and biological diversity for national security”^23,24^. All municipalities and provinces must create ERAs where ES information should inform selection. The problem is we lack standardized methods, which is impacting the consistency, credibility, and usability of ES assessments^22^.

**Fig. 1:**
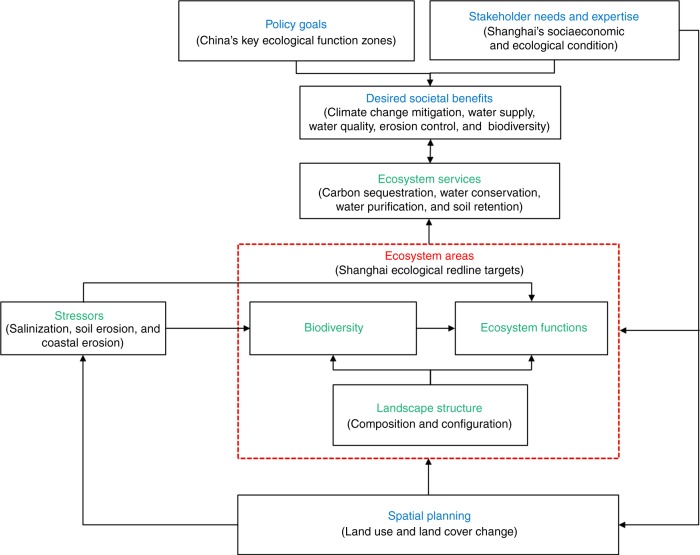
Science–policy framework linking institutional and ecological information. Ecosystem services provisioning depends on biodiversity and ecosystem functions, which are influenced by landscape structure and stressors (ecological criteria, green text). Spatial planning should manage ecosystem areas (spatial targets, red box/text) to sustain biodiversity and ecosystem functions (ecological health) by minimizing stressors and fragmentation. Ecosystem services assessments for planning should use policy goals and local stakeholder needs to select the desired ecosystem services to ground the analysis in the given decision-making context (socioeconomic criteria, blue text). Black text is the selected ecological and socio-economic criteria to determine the ecological redline areas for Shanghai Municipality. China’s Ecological Redline Policy aims to use spatial planning to safeguard ecological redlines areas (ERAs) to ensure the protection of key ecological function zones and ecologically fragile areas (vulnerable to stressors) to improve the living environment for people and biodiversity

**Fig. 2:**
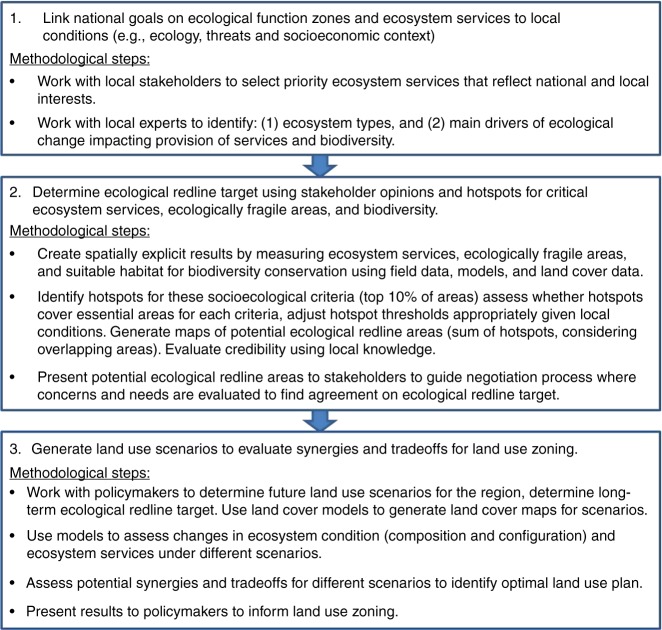
Methodological framework to conduct an ecosystem services assessment to determine ecological redline areas in China

Conceptual frameworks^1,2,5,28–33^ and spatial mapping^34–36^ have enabled progress on ES science; however, there is a fundamental lack of science–policy frameworks, explaining methodological standards for application in policy^7,37^. We create a science–policy framework (Fig. 1) that builds upon core elements of other ES frameworks (i.e., MA^1^, IPBES^29^, Ecosystem Services Cascade^38^, and Natural Capital Project^39^) but details both the specific institutional and ecological components (e.g., types of information, indicators, and methodological steps) for public policy. Here we present the transdisciplinary framework and methodology for the ERP, proposing five indicators to standardize ERA designation processes: ES hotspots; biodiversity hotspots; ecologically fragile hotspots (vulnerable to stressors); landscape structure (composition and configuration); stakeholder opinions (Fig. 2). We employ our approach to inform China’s governance process on the selection of ERAs for Shanghai Municipality. We select Shanghai as our case study because it is a priority ERP region, possessing global significance as one of the world’s most urbanized cities. The three objectives of the study are to: (1) present a science–policy framework for ES assessments applicable to ERP; (2) explain methods and ES results for determining ERAs to illustrate possible ES assessment standards; and (3) evaluate the effectiveness of ERP for reducing tradeoffs on ecosystem services.

We implement our framework to determine ERAs for policymakers in Shanghai. We first use policy goals and public preferences to select the ecosystem services. We create land use and land cover (LULC) maps to estimate ES and biodiversity, and expert opinion to determine highly vulnerable ecosystem areas to major stressors in Shanghai (known as ecologically fragile areas). We use these three indicators to determine optimal ERAs to inform a stakeholder negotiation process to determine an implementable ERA for Shanghai. To evaluate the effectiveness of the selected ERAs we compare the ERP to alternative LULC scenarios. The objective is to help policymakers select a land use plan for 2040 to inform Shanghai’s Urban Plan (2016–2040), which outlines Shanghai’s land use decisions. We compare ES outcomes under four scenarios codeveloped by policymakers and scientists: (1) baseline, current ERAs for 2014; (2) development for 2040 (no environmental constraints); (3) future ERP for 2040 (expansion of ERAs by 46%); and (4) planning for 2040 (existing ecological protection measures, except ERP). We use Markov and CLUE_S models to estimate changes in LULCs under different urbanization policies. Lastly, we compare ES synergies and tradeoffs among scenarios at local (i.e., district level) and regional (i.e., Shanghai Municipality) scales to assess whether ERP can improve ES outcomes.

From our analysis, we determine a current ecological redline target of 1098 km^2^ at the municipal scale. ERAs cover 16% of Shanghai’s total land area, representing a 174% increase in terrestrial protected area. ES criteria expand ecosystem protection by 142% (681 km^2^). Furthermore, we find ERP significantly increases ES flows compared to other land use scenarios. If properly implemented ERP could potentially reduce the tradeoff between urbanization and ecosystem protection in Shanghai.

## Results

### Ecological redline for Shanghai

As the world’s third most populous city, Shanghai embodies the sustainability challenges confronting cities worldwide; Shanghai’s experience on implementing comprehensive planning is critical to enhancing international knowledge on urban sustainability. The novel component of our approach is illustrating how scientists can use institutional information to guide the biophysical and land use modeling. Scientifically this requires understanding policies and stakeholder preferences to determine the desired human benefits to select appropriate indicators. First we select the desired ES for Shanghai by working with policymakers to determine policy goals, and conduct stakeholder surveys to assess public opinion. The government and local stakeholders want to invest in ecosystem protection/restoration to generate improvements in water resources, water quality, coastal erosion control, and climate change mitigation. The policy goals as described in Shanghai’s Urban Plan (2016–2040)^40^ are: (1) continuous water supply from freshwater sources (e.g., Qingcaosha Reservoir and Huangpu River); (2) improve water quality of major rivers for drinking water and recreation—47% of Shanghai’s freshwater ecosystems are unsafe for any use (i.e., worse than Grade V)^41^; (3) effective control of coastal erosion for land stabilization; and (4) increase carbon sequestration to offset carbon emissions (reduce peak carbon emissions by 15%) for climate change mitigation. Stakeholders select five ES: carbon sequestration, water resources conservation, water purification, soil retention, and biodiversity.

Next, we use aerial images (0.5 m) to quantify LULC for 2005 and 2014 in Shanghai Municipality to model land use patterns and ES (Supplementary Fig. 1; Supplementary Table 1). We categorize LULC into Shanghai’s five main LULC categories: (1) forest; (2) constructed land; (3) agriculture; (4) open water; and (5) beach. Second, we use the InVEST 3.2.0 models^13,39,42^ to estimate ES and biodiversity (Table 1). Ecological areas are ranked where those providing the top 10% of each ES are ES hotspots, and areas with greatest suitable habitat for biodiversity are biodiversity hotspots. Third, we gather expert opinion to rank ecologically fragile areas for key stressors: soil erosion, coastal erosion, and salinization. Ecologically fragile hotspots are the most vulnerable ecological areas to the main drivers of ecological change in Shanghai selected by local experts (see Methods). We sum hotspot areas to determine optimal ERAs to formulate a planning map (resolution 50 m). Subsequently local governments facilitate a stakeholder negotiation process using scientific maps on optimal ERAs to guide discussions to build consensus on an implementable target.

**Table 1:** Indicators and data to determine the ERAs for 2014 in Shanghai Municipality

Criteria	Indicators	Main data
Ecosystem services hotspots	Carbon storage and sequestration (carbon sequestered tonnes)	LULC, biomass values, and net primary production
	Water storage (water retained m^3^)	LULC, rainfall, runoff, and evapotranspiration
	Water purification (nitrogen removal kg)	LULC, digital elevation model, pollution export, and filtration coefficients
	Soil conservation (soil retained tonnes)	Digital elevation model, sediment retention value, soil characteristics, rainfall, vegetation cover, and management factor
Ecologically fragile hotspots	Soil erosion (tonnes)	Digital elevation model, soil characteristics, rainfall, vegetation cover, and management factor
	Desertification (dimensionless)	Humidity index, number of days with wind speed >6 m s^−1^, soil texture, vegetation cover, and evaporation/rainfall
	Salinization (dimensionless)	Groundwater mineralization and topography
Biodiversity hotspots	Habitat quality (dimensionless)	Sensitivity of habitat types to each threat, relative impact of each threat on habitat, and distance of habitat from stressor
Stakeholder opinions	Stakeholder preferences for different ecosystem services (percentage)	Survey data

Note: InVEST models were used to estimate the ecosystem services and biodiversity hotspots; delphi method used to estimate the ecologically fragile areas; survey was conducted to determine stakeholder preferences for different ecosystem services.

**Fig. 3:**
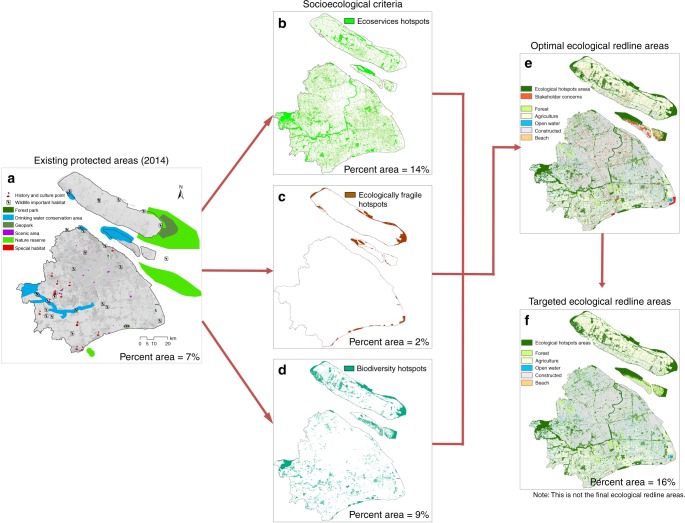
Spatial results for each step of the spatial planning process to select the ecological redline areas for Shanghai Municipality. **a** Existing protected areas for 2014, small patches with limited connectivity, representing only 7% of Shanghai’s land area. We used the Ministry of Environmental Protection criteria to formulate three indicators for determining optimal ERAs: **b** ecosystem services hotspots; **c** ecologically fragile hotspots; and **d** biodiversity hotspots. Next optimal ERAs (determined from socioecological criteria) were presented to stakeholders who refined ERAs. **e** Stakeholders indicated areas of concern meaning areas they felt were not manageable (i.e., removal of small patches) or pose serious social and economic burdens to communities (i.e., removal of major disagreement areas). **f** Targeted ERAs are not the final ERAs but Shanghai Municipality is considering using them to form the final ERAs. The maps in this figure were made by the author in ArcGIS software for use in this paper

We determine 929 km^2^ of the land area in Shanghai is ES hotspots, consisting of Shanghai’s key drinking water sources (e.g., Huangpu River, Qingcaosha, Chenhang, and Dongfengxisha) and major forest patches (e.g., Dongping Forest Park, Haiwan Forest Park, and Gongqing Forst Park). Next, we estimate 171 km^2^ of Shanghai’s land area is ecologically fragile hotspots, representing the most threatened habitats in Shanghai, mainly located on Chongming Island due to sea-level rise (e.g., Dongtan Wetland Park and Xisha Wetland Park). Furthermore, we determine 643 km^2^ is biodiversity hotspots, which encompass major wetland reserves, coastal marshes, bird sanctuaries, etc. Subsequently we evaluate whether the 10% threshold for hotspots represent the majority of ES production, vulnerable areas, and biodiversity areas. We calculate ES hotspots represent 35% of estimated carbon sequestration; 49% of estimated water conservation; 33% of estimated water purification; and 42% of estimated soil retention. Ecologically fragile hotspots represent 8% of expert selected vulnerable areas, and biodiversity hotspots represent 15% of estimated suitable biodiversity habitat (Supplementary Fig. 2). For spatial planning, we have to balance space constraints with the greatest potential to improve environmental quality for human welfare given the policy goals. Hotspot indicators, in particular ES hotspots, seem to strike this balance for expanding the consideration of multiple environmental benefits in the spatial planning process in Shanghai. Lastly, we combine these three spatially explicit layers (Fig. 3b–d) subtracting 470 km^2^ of overlapping areas to produce the optimal ERAs, totaling 1273 km^2^.

The optimal ERAs established through this analysis were presented as a part of a stakeholder negotiation process led by the Shanghai Municipal Government (SMG), Shanghai Environmental Protection Bureau (SEPB), and Shanghai Municipal Planning and Land Resources Administration (SPLRA). The stakeholder groups are district and township governments, enterprises, farmers, and residents that are likely to be impacted by the ERAs. There are two types of consultations: (1) district-level scoping and (2) public comment. For the district workshops, the aim is to obtain agreement on the location of ERAs at the district level. The SEPB and SPLRA presented the spatial maps to district governments and other key agencies then we discussed the proposed ERA spatial distributions, ERA criteria, and our methodology. Coproducing the ES information with the SEPB was critical for legitimizing the ES analysis (see Supplementary Methods for details on the stakeholder engagement; Supplementary Table 2 defines the stakeholder groups). Disagreements mainly concern the prevention of future development activities in certain areas. District leaders refined the optimal ERAs using local expertize to determine manageable ERAs (i.e., elimination of small patches). The SMG plans to financially compensate impacted stakeholders to offset economic losses associated with relocation and prohibition of development near ERAs; currently the financial compensation program is undergoing development. Preliminarily, the stakeholder negotiation process removed 176 km^2^ of optimal ERAs (Fig. 3e). Scientifically, we approve of this reduction because it removes mainly isolated patches of forest and grassland; it represents the lowest estimated impact on ES and biodiversity given stakeholder concerns. The current selected ERAs encompass a total protected area of 1098 km^2^ (142% increase in protected areas from 2014), accounting for 16% of Shanghai’s total land area (Fig. 3f). Please note these are not the final ERAs but our analysis is being used as part of the process to form Shanghai’s final ERAs. The political process will take time and several rounds of negotiation, which is beyond the scope of our analysis . To date ERAs represent China’s strictest environmental protection standard. According to China’s Environmental Protection Law (2015), ERAs must incur no ecological degradation and no reduction in acreage, the government can only increase acreage overtime^22–24^. If properly implemented ERAs for the first time would protect the majority of Shanghai’s terrestrial ecosystems. Prior to ERP, the main ecosystem types under protection were drinking water sources, wetlands, and marine reserves typically determined using single criteria (Fig. 3a). The ERP using ES criteria leads to a spatial plan that for the first time coordinates ecological protection across terrestrial ecosystems aimed at multiple social benefits. From the stakeholder negotiation process, we determine stakeholders came to agreement on ERAs using the ES assessment because the central government is now prioritizing the enforcement of ecosystem protection to sustain ES.

### Future land use plan

**Fig. 4:**
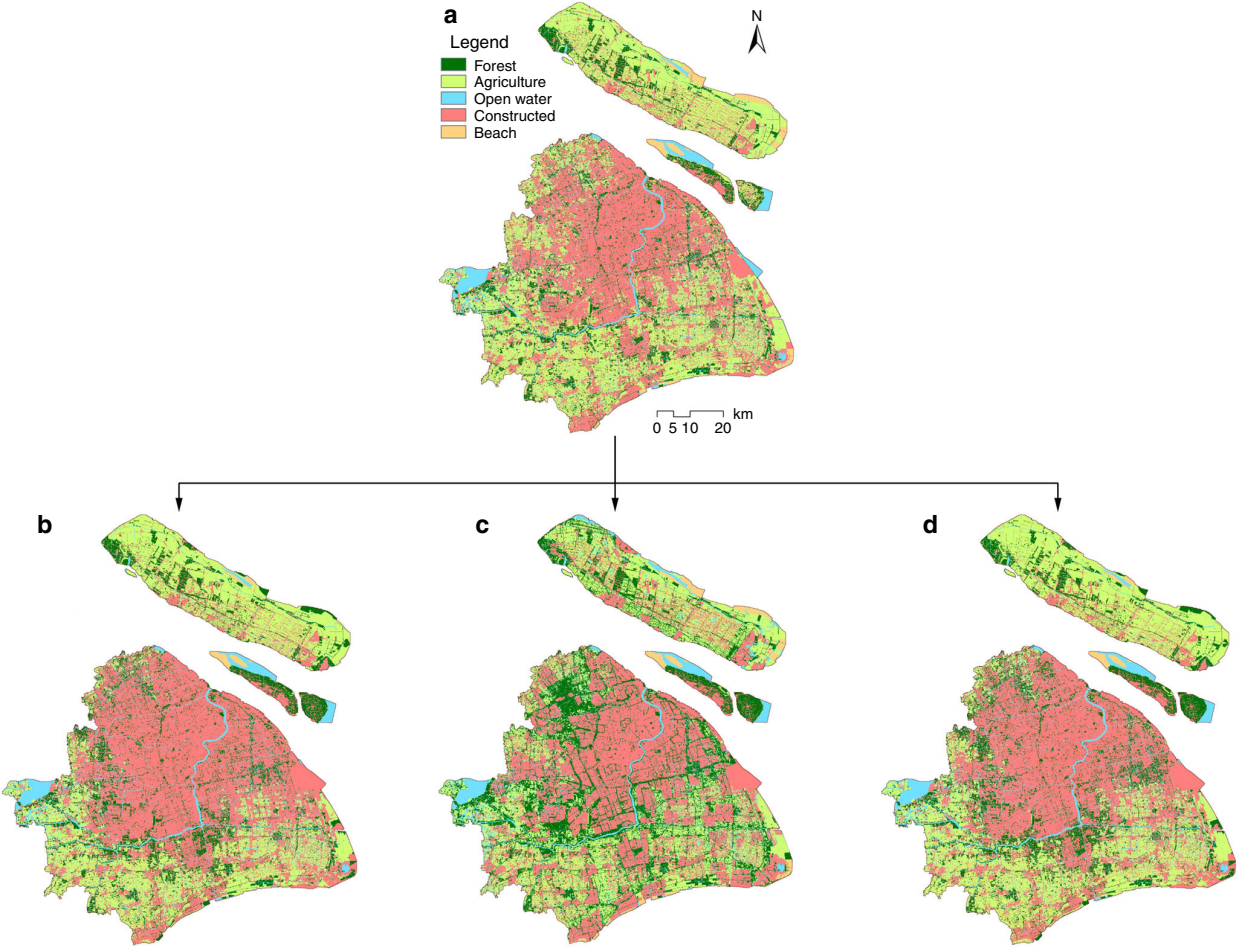
Planning scenarios under different environmental and development policies for Shanghai. **a** Baseline scenario using current LULC with ERAs in 2014 (S1). **b** Development scenario for 2040 with no ERP and no policy constraints (S2). **c** Future ERP scenario for 2040 representing planned expansion of ERAs by 501 km^2^ (S3). **d** Planning scenario for 2040 where existing ecological protection policies are implemented, except ERP. The maps in this figure were made by the author in ArcGIS software for use in this paper

**Fig. 5:**
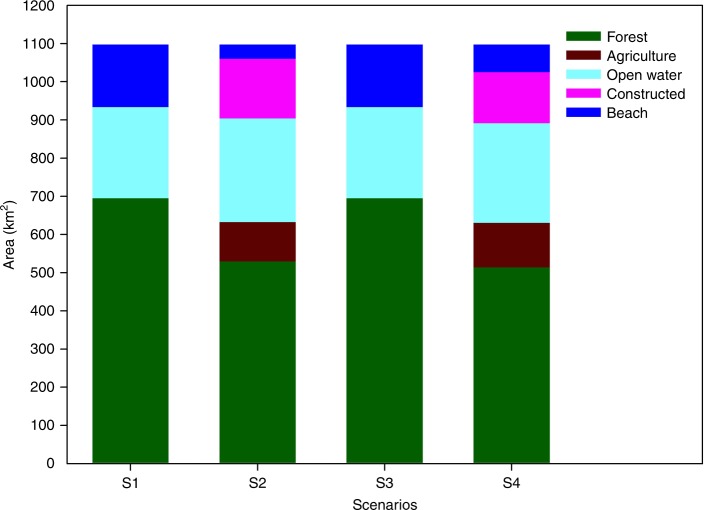
Ecosystem composition of ERAs under four alternative scenarios. Shown in different colors are the total estimated land areas (km^2^) of each major land cover and land use type in the ecological redline areas (ERAs). The ERAs in scenario 1 and scenario 3 are mainly composed of forest, open water, and beach. Without the ERP constraint, we estimate the ecosystem composition of the ERAs change, shown in scenarios 2 and 4 where forest area and beach are reduced due to land use conversion to agriculture and constructed lands

ES assessment for spatial planning should help clarify tradeoffs between development and environmental protection to promote wise management that fosters synergies and reduces tradeoffs^1,2^. We explore three alternative future scenarios for 2040 (Fig. 4) derived from the Markov and CLUE_S models, which were codeveloped with policymakers and urban planners for the SMG and Shanghai Development and Reform Commission. We compare the spatial configuration and ES outcomes among the current ERAs (S1), future development scenario (S2), future ERP scenario (S3), and future planning scenario (S4) in 2040 (Table 2). Scenarios have different impacts on the ecosystem composition and spatial distribution of the landscape (Fig. 4; Supplementary Table 3). In S2 constructed area increases by 15% compared to S1 mainly from the conversion of agriculture and open water. S2 continues the conventional urbanization trend in Shanghai. In S4 constructed area increases by 3% from S1, however, in S3 constructed area decreases by 10% from S1. Forest area increases under all future scenarios (S2–S4) when compared to S1. This result is consistent with the LULC changes in recent years in Shanghai due to increased afforestation efforts^43^. In S2 and S4 the increase in forest area is due to future afforestation efforts, however, existing forest area decreases (Supplementary Fig. 3). An increase in young secondary forests, however, may not guarantee the same quality and/or quantity of ES as more mature forests. Forests and agriculture retain most of their original distributions in S3 where 92% of forests and 81% of farmlands are unchanged. Forests in S2 and S4 retain 68% and 86% of their original areas, while agriculture retains 71% and 75%, respectively (Supplementary Fig. 4). The largest difference in ecosystem composition between the future scenarios is the reduction in beach area between S3 and S2 and S4. S3 is the only future scenario that retains open water and beach areas at current levels (Fig. 5).

**Table 2:** Development policies describing urbanization rates and spatial form based on various urban planning policies

Scenario	Planning and environmental policies	Spatial changes
Baseline 2014	Current land use and land cover with targeted ecological redline areas (ERAs) for 2014, which are ERAs from the analysis.	(1) Constructed land = 42%, (2) agriculture = 35%, (3) forests = 12%, (4) open water = 9%, and (5) beach = 2%
Development 2040	No implementation of Ecological Redline Policy (ERP), and no new policies to constrain growth. Uncontrolled urbanization with population size of 31 million (25% total growth rate) and 5% GDP growth rate for 2040.	(1) Constructed land = +6%, (2) agriculture = −7%, (3) forest = +3%, (4) open water = −1%, and (5) beach = −1%
Ecological Redline Policy 2040	Expansion of ERAs by 501 km^2^ by: (1) planting vegetation buffers along river banks and (2) transforming industrial and agricultural areas to forests (afforestation). Condensed urbanization with projected population size of 25 million (4% total growth rate) and 5% GDP growth rate for 2040.	(1) Constructed land = −4%, (2) agriculture = −4%, (3) forest = +9%, (4) open water = 0%, and (5) beach = 0%
Planning Scenario 2040	Implementation of existing ecological protection policies outlined in Shanghai’s Urban Plans (1999–2020; 2016–2040), excluding the ERP. Projected population size of 25 million (4% total growth rate) and 5% GDP growth rate for 2040.	(1) Constructed land = +1%, (2) agriculture = −4%, (3) forest = +5%, (4) open water = 0%, and (5) beach = −1%

We also calculate connectivity index (CI) and fragmentation index (FI) values to evaluate the degree of connectivity between ecosystem types known to influence ecosystem functionality and biodiversity; each scenario has different ecosystem configurations compared to S1 (Supplementary Fig. 5). For S2 the FI value is 13.65 km^−^^2^ (16% decrease from S1), while the CI value is 90.16 (4% increase from S1). In comparison, FI for S3 is 11.27 km^−2^ (31% decrease from S1) and CI is 95.73 (11% increase from S1). The increase in CI and decrease in FI values in S3 from S1 are due to the establishment of ecological corridors to form riparian buffers along Shanghai’s major rivers like Huangpu River and Suzhou River. Our results suggest if properly enforced, the ERP is the only urban plan that can maintain ecosystem composition by protecting forest, open water, and beach areas while minimizing agricultural losses (Fig. 5).

### Ecosystem services

**Fig. 6:**
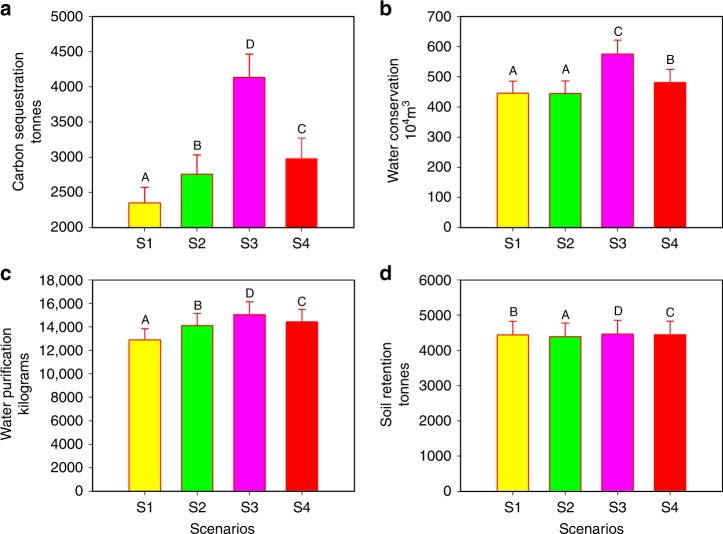
Ecosystem service values for each scenario. **a** The estimated average annual carbon sequestration service in tonnes of carbon stored for each scenario. **b** The estimated average annual water conservation service in 10^4^ m^3^ of water stored for each scenario. **c** The estimated average annual water purification service in kilograms of nitrogen stored for each scenario. **d** The estimated average annual soil retention service in tonnes of soil retained for each scenario. For each ecosystem service, we estimate the annual production at the subdistrict level (*N* = 236 subdistricts) to determine the average annual amount; data show mean ±  standard errors of each service calculated. The different colors in each graph represent the different scenarios where S1 is yellow, S2 is green, S3 is magenta, and S4 is red to illustrate the tradeoffs among land use plans on the suite of ecosystem services. The different uppercase letters denote significant differences among scenarios at *P* < 0.01 in a paired samples *t* test

Shanghai wants to enhance ES levels overtime, despite expected future population growth, but performance differs among the scenarios. Overall, S3 has the highest estimated ES production compared to all scenarios. We estimate the future ERP will incur fewer ES tradeoffs with development compared to other land use policies (Fig. 6). We find a strong positive correlation among all four ES for each scenario (Pearson correlation; d*f* = 234, *P* < 0.01) (Supplementary Table 4). When one ES significantly increases from S1 to S4, we find the other three ES also significantly increase across S1–S4 (Fig. 6). We also find S3 has the highest landscape connectivity and lowest fragmentation, and has significantly greater ES levels compared to all other land use scenarios (paired samples *t* test; d*f* = 235, *P* < 0.01). We find statistically significant ES tradeoffs between S3 (i.e., future ERP) and S2 (i.e., development) (paired samples *t* test; d*f* = 235, *P* < 0.01). If the ERAs are not implemented and Shanghai continues to pursue conventional urbanization policies, we estimate Shanghai will receive: 0.33 million tonnes less carbon sequestration; 0.31 billion m^3^ less water supply; 0.22 million kg less nutrient removal; and 0.02 million tonnes less coastal erosion control (Fig. 6). We also observe a statistically significant increase in ES in S3 compared to S1 (i.e., current ERP) since the main objective of the future ERP is to enhance ecosystem functionality through restoration. From the baseline scenario, we estimate S3 increases: carbon sequestration by 0.42 million tonnes; water retention by 0.30 billion m^3^; nutrient removal by 0.50 million kg; soil retention by 0.01 million tonnes.

**Fig. 7:**
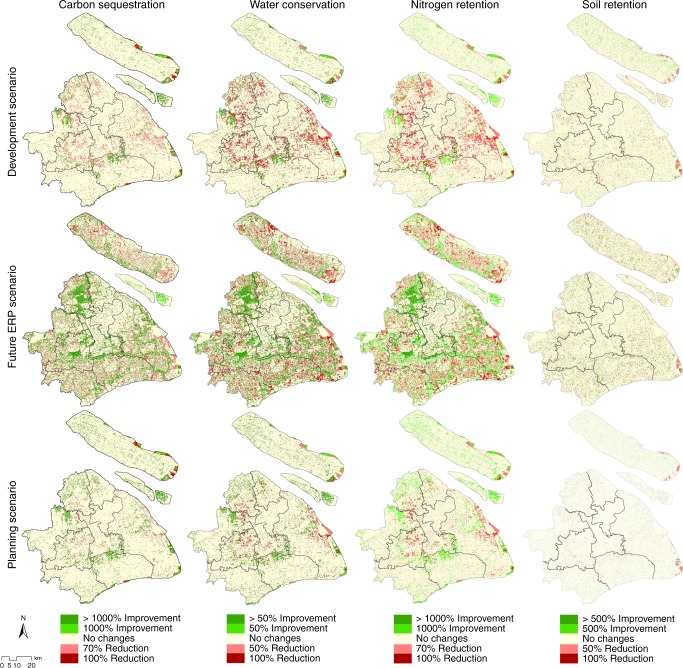
Performance of the Ecological Redline Policy. The horizontal panels represent the change in each ecosystem service relative to the baseline scenario (i.e., scenario 1), and the vertical panels represent the performance of the different future scenarios. The data show the percentage of improvement (green) or reduction (red) of each ecosystem service in each scenario relative to the baseline scenario. The maps in this figure were made by the author in ArcGIS software for use in this paper

However, synergies and tradeoffs on ES production shift when evaluating ES production at the local scale (i.e., district level). Locally, the most dramatic improvements and reductions in ES occurr in S3. S3 has the most patches with greater than 1000% improvement in carbon sequestration and nitrogen retention, and greater than 100% improvement in water retention (Fig. 7). However, substantial improvements in ES production in the ERAs lead to the highest estimated (70–100%) reductions in the same ES in southern districts. From the modeling, we observe a tradeoff at the local scale: the expansion of ERAs by increasing forest area causes increased development in southern districts. Policymakers project Shanghai’s population will grow by 4% from 2014 to 2040. Under this condition, achieving more ecosytem protection causes future development to increase in southern districts. Therefore an important future research topic is evaluating whether the spatial alterations in the ES production translate to tradeoffs in the spatial distribution of ES beneficiaries. To evalute ES outcomes and human welfare improvements will require monitoring to: (1) find ways of matching ES supply and ES demand (i.e., minimize the unequal distribution of benefits) and (2) determine ways of managing ERAs and other land uses to meet Shanghai’s ES goals.

## Discussion

Our work provides new insights on integrating ES science into urban planning by illustrating the various methodological steps of the science–policy process from design to application in policy. Currently, we lack science–policy examples of ES application in policy^8,44^. A core objective of the ES approach is to integrate ecosystem management into development decisions to promote sustainability^5^, however, the majority of academic recommendations on the advantages of ES assessment are theoretical with minimal evidence of ES assessment leading to more comprehensive planning^7,21^. A key reason for the science–policy disconnect is that the majority of ES studies and assessments focus on biophysical and/or monetary accounting with minimal integration into a social (decision-making) process^6^. In our study we implement a framework that details how to incorporate the needs of stakeholders in particular policymakers into the development of the ES science, and illustrate how policymakers can use the science in the policy process. Our findings suggest that including ES criteria into the urban planning process doubled ecosystem protection in Shanghai. Stakeholders approved of the expansion of ecosystem protection because ES information clarified how Shanghai’s ecosystems support various societal goals. We found the key is clearly relating ES information (ecosystem components and human benefits) to the given institutional context by having local policies guide the ES assessment framework. Scientists need to relate ES categories and indicators to specific policy goals (targets). For spatial planning, managers want credible and legitimate spatial targets. Therefore we need to craft an interdisciplinary science that relates stakeholder needs (wants) to core ecological measurements across space and time.

Since 1997 the number of scientific publications addressing ES has increased by near 30-fold, yet interdisciplinary assessments make up less than 9% of ES studies^45^. A major challenge is unifying fragmented disciplines to address real-world problems. In our experience, we are finding a critical first step is formulating practical interdisciplinary frameworks to organize indicators and methodologies. The problem is the majority of existing ES frameworks are general because they lack connections to actual decision-making contexts^46,47^. Not engaging with decision-makers has led to a proliferation of frameworks and methods that lack cohesion, which is limiting scientific progress^47^. Our work addresses this specific gap since we utilize the ERP to develop an approach for China’s regulatory context in order to explore methodological steps of moving the science toward ES standards. We recognize there are limitations to our ES measurements since we were unable to create ecological production functions^37^ given China’s policymaking timeline. Despite limitations in the ES measurements, we address the fundamental dilemma of whether ES standards (e.g., ES assessment for ERAs) can inform urban planning. Overtime, our scientific capabilities on ES measurements will improve, but better data alone will not guarantee relevance for policy. Our work shows scientists must explicitly consider the institutional context to advance the science and practice of ES assessment in urban planning.

The ES approach can help organize core policies into societal needs/wants to clarify how ecological changes associated with different actions may impact desired goals. We use China’s national EFZs and the Ministry of Environmental Protection’s general guidelines on ecological redlines (Supplementary Table 5). China currently has a unique decision-making context since the government wants ES information to select ecological redlines. However, the lack of ES frameworks for policy has led to confusion and inconsistency across China. Having a decision-making context where institutions need ES information should accelerate the use of ES information, however, we are learning that the organization of information and the type of information remain critical for effective application. Many ES assessments have been conducted in China, but few have led to local policy changes, despite the central government’s mandate on implementing the ERP. On February 14, 2017 the SMG officially announced it will implement the ERAs, placing over two-fifths of its land area and 1000 km^2^ of surrounding waters into “no development zones” to preserve ecological functionality^48^. Our assessment is not the final ERAs, but the work presented in this study represents a stage of the policy process to establish final ERAs. Shanghai officially released its preliminary ERAs on June 28th, 2018. Also urban planners are using the ERP scenario information to inform the creation of ecological corridors to meet the 2040 ERA target. The objective is to increase ecosystem connectivity by reducing structural fragmentation since current ERAs are small patches. Full implementation of the ERAs will take time since Shanghai has to find innovative political solutions to reconcile serious tradeoffs. Doubling the amount of ecosystem protection will require major land-use shifts.

From our experience, we identify four key lessons on the science–policy process for integrating ES information into urban planning. First governments likely are unable to implement individualized ES standards for a diversity of ES. In the case of China, the Chinese Government has been trying for over a decade to implement EFZs for different ES. However, managers need clear spatial targets to manage large landscapes. Thus, we believe a plausible pathway on ES standards is establishing ES assessment protocols to evaluate the provision of multiple ES from an ecosystem area (spatial configuration) target. We evaluate the ecosystem area target in terms of ES criteria where the evaluation process has been standardized on evaluating ES. Second, scientists need to develop frameworks outlining core socioecological criteria for determining ecosystem area targets. We need clear illustrations of the main methodological steps for ES assessment to communicate the importance of different socioecological components to stakeholders. In our assessment we select five criteria: (1) ES hotspots, (2) ecologically fragile areas, (3) biodiversity hotspots, (4) landscape structure, and (5) stakeholder opinions. Third stakeholder engagement and negotiation are critical for implementing spatial targets. The SMG oversaw the stakeholder negotiation process, but we worked closely with policymakers to develop and refine ES maps to inform ongoing negotiations. Furthermore, we worked closely with urban planners to develop feasible scenarios where we utilize official urbanization projections and spatial planning regulations to assess ES tradeoffs. Lastly, for general planning we are finding basic ES measurements are useful, but empirical measurements linking ecosystem structure and functions to human benefits are needed. Moving from planning to practice requires monitoring; the challenge is measuring ES using ecological production functions to link intermediate and final ecosystem services^37^. The next step is to develop empirical datasets to link ecosystem characteristics and human welfare benefits to evaluate the socioecological outcomes from the ERAs to refine actual actions on the ground.

In conclusion, our study supports the use of ES information in urban planning for developing more comprehensive plans on ecosystem protection. Currently planning policies and actions are informed by data with low temporal and spatial resolutions^21^. Our study supports the hypothesis that ES assessment can help policymakers generate more comprehensive spatial plans. Moreover, we develop a new framework for the ERP, which we hope can advance domestic efforts in China as well as similar efforts internationally. Ultimately, our study helps provide evidence that ES assessment can enhance urban planning when scientists integrate institutional and ecological components together.

## Methods

### Selection of ecosystem services

An ES assessment was conducted to determine an ecological redline for Shanghai Municipality, and assess the potential effectiveness of ERP for enhancing ES. We worked with government officials and surveyed the public to determine priority ES for the assessment. First, we worked with the SEPB to link local and national policy goals under the ERP. Second, we surveyed Shanghai residents to determine public preferences and expectations from increased green space via the ERP. The questionnaire featured demographic questions and ES questions in Mandarin. We conducted the questionnaire from July 1 to August 1, 2014 (*N* = 849). We asked participants to select the ES they felt most important to them and Shanghai. The questionnaire consisted of 21 questions divided into four sections, including single choice, multiple choice, and open-ended questions. We designed the first section to determine public perceptions of urban green space in Shanghai. For example, we asked residents how often they visit local parks, and their opinions (satisfaction and problems) of current green space in Shanghai. The second section contained questions on public expectations of ES. The third section asked for sociodemographic information: gender, age, occupation, and income. We crafted the fourth section to determine the distribution of beneficiaries of urban green space. We used random selection to determine ten urban parks in Shanghai to conduct in-person interviews. The total number of respondents was 849. For each respondent we collected demographic information, such as gender, age, family size, education, occupation class, location of residence, and income (see Supplementary Methods for details on the social survey).

We used the questionnaire to determine the priority ES to the public in Shanghai. We asked surveyors: “In your opinion, which of the following ecosystem services is the most important for Shanghai and you (single choice)?” We listed the following ES choices: (1) carbon sequestration, (2) water quality regulation, (3) water resources conservation, (4) soil conservation, (5) biodiversity conservation, (6) leisure and recreation, (7) flood regulation, and (8) other. Before introducing the question, we gave a brief explanation of each ES and how they relate to people’s daily lives. The top three ES were: water purification, water resources conservation, and soil conservation. Lastly, we related the top ES of policymakers with the top ES of surveyors to strike a balance between policy goals and public demand. The final selected ES are: (1) water resources conservation; (2) water purification; (3) carbon sequestration; (4) soil conservation; and (5) biodiversity conservation. For the ecological redline analysis we categorize the first four as the ES criteria, and biodiversity conservation as the biodiversity criteria.

### LULC analysis

Multitemporal aerial images (spatial resolution = 0.5 m) were taken by helicopters to determine LULC information for 2005 and 2014. We obtained 41 images from January to March, 2005 and January to March, 2014. The images were merged together in ERDAS Imagine 9.3, and manual visual interpretation via ArcGIS 10.0 was used to delineate polygons for the five LULC classes: (1) forest; (2) agriculture; (3) constructed land; (4) open water; and (5) beach. The overall classification accuracy is 94% for the 2014 LULC map (Supplementary Methods for more details; Supplementary Table 6). The LULC layers were converted to a grid format with a spatial resolution of 50 m to conduct the ecological redline and scenario analyses.

### Ecological redline analysis

First we performed the scientific analysis to identify an optimal ecological redline target for Shanghai using three indicators: (1) ES hotspots, (2) ecologically fragile hotspots, and (3) biodiversity hotspots. Hotspots have been widely used for identifying priority areas for biodiversity conservation, and scientists are extending the concept to identify priority areas for ES and vulnerable areas. Hotspots are areas of critical management importance since they are critically threatened, high in ES production, and high in biodiversity^49^. The Supplementary Methods contains a detailed explanation on our selection of the indicators. For each of the three hotspots, we selected areas representing the top 10% of each indicator^49,50^; we verified this threshold relating each hotspot to the total estimated ES production, total estimated vulnerable areas, and total estimated suitable habitat for biodiversity (Supplementary Fig. 2). The total optimal ERA was the sum of the hotspot areas, considering overlapping areas. Second, we engaged in a stakeholder negotiation process among local governments, scientists, and residents (Fig. 3e). Stakeholders first reviewed the optimal ERAs and identified manageable areas (i.e., removal of small patches) then removed areas of serious disagreement. The spatial planning process for ERAs was an iterative, co-development process. Local governments reported to the SEPB who reported to scientists where we assessed the impact of stakeholder concerns on the ES, ecologically fragile areas, and biodiversity. We reported to the SEPB at least five times per year; the SPLRA and SEPB used the scientific information to guide the stakeholder visioning and negotiation on ERAs.

Ecosystem services hotspots were estimated using the InVEST 3.2.0 models, which is a GIS-based method for estimating ES across a landscape, given different LULC scenarios^13,28,42^. We calculated carbon storage using the InVEST carbon storage and sequestration model to estimate aboveground biomass, belowground biomass, soil, and dead organic matter per LULC type. Carbon sequestration was evaluated as net primary production based on photosynthesis process. We parameterized the model using biomass values (Supplementary Table 7) from studies in Shanghai, and the Intergovernmental Panel on Climate Change Guidelines for National Greenhouse Gas Inventories^51^. We compared our carbon sequestration values with the empirical carbon sequestration values for Shanghai in the literature^52,53^. We found no significant difference between our estimates for carbon sequestration and the literature values (*t* test; *t* = −0.96, d*f* = 8, *P* = 0.36).

Water resource conservation is defined as the ability of ecosystems to intercept or store water resources from precipitation, which is calculated by precipitation minus evapotranspiration and runoff. First, we estimated precipitation minus evapotranspiration by using the water yield model in InVEST. Water conservation was then evaluated as water yield minus runoff. The InVEST model estimates the relative contributions of water from different parts of the landscape to evaluate how possible changes in land use patterns could impact the annual surface water yield. The model does not differentiate between surface, subsurface and baseflow, but assumes water yield from a pixel reaches the point of interest via one of these pathways. We derived input values using local data on rainfall, runoff^54^ and ET coefficients^39^ (Supplementary Table 8). Hamel and Guswa^55^ found the InVEST water yield model was able to represent differences in land uses and the spatial distributions of water provisioning. The most important model parameters for reducing model errors are climate variables in particular annual precipitation. We obtained annual precipitation from 11 monitoring stations in Shanghai^54^. Our modeled water yield value was 3.02 billion m^3^ similar to the observed value of 2.28 billion m^3^ in 2013^54^.

Water purification was calculated using the InVEST nutrient retention model to estimate the amount of nitrogen retained in the landscape based on runoff, digital elevation model, soil characteristics, and the pollution export, and filtration coefficients^56^ linked to different LULC types (Supplementary Table 9). InVEST estimates the contribution of vegetation and soil to water purification through the removal of nutrient pollutants from runoff. The model assumes that non-point sources of water pollution result from export that can be mitigated by terrestrial vegetation. We used discharge of dissolved nitrogen as our proxy for pollution. The final model output is the total nitrogen retention (kg y^−1^) for each grid cell. We compared our model results to four studies that measured nitrogen retention for Taihu River Basin where Shanghai is located. Measured nutrient retention values ranged from 1.56 to 7.27 ton km^−2 (^^57–59^^)^; our modeled value was 2.02  ton  km^−2^, which falls within the range of reported observed values.

Soil retention was calculated using the InVEST sediment delivery ratio model as the average annual amount of soil loss from each parcel of land. InVEST uses the Universal Soil Loss Equation to identify the land parcel’s potential soil yield and capacity to retain sediment^60^. Input data is DEM, management practices, sediment retention value, vegetation cover, and management factor per LULC type (Supplementary Table 10). Management factor reflects the impact of soil and water conservation measures (e.g., terraced fields and cement protective walls) on soil retention. Hamel et al.^60^ found the InVEST soil loss model is useful for first order assessments of sediment dynamics. Shanghai is a highly urbanized watershed, thus we expect low soil loss values due to the high percent impervious surfaces. We compared our model values to measured soil retention in the Taihu River Basin. Soil retention values from the literature were 1.11^58^ and 0.79 thousand tonnes km^−2^ yr^−1^^61^ similar to our value of 0.70 thousand tonnes km^−2^ y^−1^.

We calculated ecologically fragile hotspots by first estimating the sensitivity of Shanghai’s ecosystems to three key external disturbances (i.e., main drivers of ecological change): soil erosion, desertification, and salinization. We created a scoring matrix for each disturbance using expert consultation based on the Delphi method then classified each indicator into five levels using average scores (Supplementary Tables 11 and 12). We defined areas with the highest sensitivity as ecologically fragile hotspots (top 10% of most sensitive areas to the selected disturbances).

We calculated biodiversity hotspots using the InVEST model for habitat quality, which estimates the extent of suitable habitat for organisms by combining information on LULC suitability and threats to biodiversity. This approach generates information on the relative extent and degradation of different habitat types in a region. The model is based on the hypothesis that areas with higher habitat quality support higher richness of native species, and decreases in habitat extent and quality lead to reductions in species persistence. Habitat quality is estimated as a function of four factors: (1) relative impact of each threat; (2) relative sensitivity of each habitat type to each threat (Supplementary Table 13); (3) distance between habitats and sources of threats (Supplementary Table 14); and (4) degree of legal protection. Terrado et al.^62^ found InVEST estimates for biodiversity richness are useful surrogates for biodiversity in terrestrial and aquatic ecosystems; modeled habitat quality correlated with biodiversity at the river basin scale.

The stakeholder negotiation process was led by the SEPB and SPLRA who oversaw an iterative process of scientific analysis, stakeholder visioning, review/negotiation, and ERP development. The SPLRA first invited relevant stakeholder groups to participate in the scoping discussions (i.e., district governments and scientists). SPLRA established an interdisciplinary team of scientists, consisting of specialists from the Shanghai Academy of Environmental Sciences, Shanghai Urban Planning and Design Research Institute, Shanghai Municipal Institute of Surveying and Mapping, and the Shanghai Ocean Planning and Design Research Institute, etc. We worked collaboratively with SEPB and SPLRA to conduct the ecological redline analysis to determine the optimal ERAs. There are two types of consultations: (1) district level and (2) public comment. At the district level workshops SEPB and SPLRA presented the spatial maps to district governments and scientists. Together we discussed our selection of  the ERA criteria, our methodology, and the results (Supplementary Table 2 and Supporting Materials describe the stakeholder groups in detail). Local governments reported stakeholder visioning, expertise, and concerns to the SEPB and SPLRA who reported stakeholder input to us. We utilized the stakeholder information to refine the scientific analysis. Subsequently the scientific information was reported to the SEPB and SPLRA who negotiated with stakeholders to find a balance between their concerns and securing ES, ecologically fragile areas, and biodiversity.

### Land use scenarios

We worked with policymakers and urban planners to determine development scenarios for the SMG and Shanghai Development and Reform Commission. The SMG was developing Shanghai’s Urban Plan (2016–2040), thus policymakers wanted information, comparing baseline ERAs to different land use scenarios to determine the best approach for improving the environment. First the SMG predicted future economic and population growth rates working with scientific and policy research teams (e.g., Fudan University, Shanghai Academy of Social Sciences, Shanghai Tongji Urban Planning and Design Institute) using past population trends to generate future population projections^40^. They generated linear regression models considering population growth factors (e.g., historical birth and death rate per age group, degree of education, immigration and migration rates) and GDP growth rate. Next, we refined the scenarios using different spatial planning policies for regulating urban spatial form defined by policymakers. We created one baseline scenario and three alternative future scenarios (Fig. 5; Table 2):

Scenario 1 (S1) is ERP baseline using current LULC with targeted ERAs for 2014.

Scenario 2 (S2) is development scenario with no ERP implementation for 2040 and no policy constraints on development. This scenario is characterized by uncontrolled urbanization with projected population size of 30.69 million (25% total growth rate) and 5% GDP growth rate in 2040.

Scenario 3 (S3) is future ERP scenario for 2040 is where we worked with policymakers to increase ERAs by 501 km^2^, representing optimal areas for enhancing ecosystem connectivity among natural and seminatural habitats. This scenario is characterized by condensed and slower urbanization in which population size is limited to 25 million (4% total growth rate) and 5% GDP growth rate in 2040. The main LULC difference between S3 and S1 is the expansion of ERAs: (1) vegetation buffers along river banks (main action); (2) transforming industrial areas to forests (afforestation); and (3) transforming agricultural areas to forests (afforestation).

Scenario 4 (S4) is planning scenario for 2040 considers existing ecological protection measures in Shanghai’s Urban Plans (1999–2020 and 2016–2040), excluding the ERP. This scenario is characterized by condensed and slower urbanization in which population size is limited to 25 million (4% total growth rate) and 5% GDP growth rate in 2040. Also, S4 and S3 both implement the permanent farmland policy and urban boundary policy, which are aimed at reducing cropland losses.

We forecasted different LULCs for alternative scenarios using the: (1) Markov model to estimate areas of different land-uses in 2040 and (2) CLUE_S model to estimate spatial patterns in 2040 (Supplementary Fig. 6). For the Markov model, we generated a transfer area matrix and transfer probability matrix for 2005–2014 (Supplementary Table 15). The Kappa coefficient was used to validate the simulation results where the Kappa coefficient was 0.88, indicating suitable simulation results. We used the Markov model to predict LULC areas under S2, and Shanghai’s Urban Plan (2016–2040) to estimate LULC areas under S3 and S4.

Next, we created logistic regression models to determine the relationship between various LULC types (response variables) and drivers of LULC change (explanatory variables) to forecast the spatial distribution of LULCs. We selected seven known drivers: (1) GDP, (2) population density, (3) distance to main rivers, (4) distance to roads, (5) distance to railways, (6) distance to ports, and (7) distance to airports. Population and GDP were collected from the Shanghai Statistical Yearbook and Shanghai’s Urban Master Plan (2016–2040); the other factors were calculated in ArcGIS 10.0 using the spatial analysis module. We only set one LULC rule for S3 where the established ERAs in S1 are not changed in S3; no other rules were set. LULC types can transform freely from one type to another. We generated the logistic regression models to determine the probability of occurrence of each LULC type in a particular grid (Supplementary Table 16). We used the Relative Operating Characteristic value (ROC) to test the significance of the logistic regression models. ROC values were greater than 0.7 (Supplementary Table 18), indicating model suitability for predicting and simulating the spatial pattern of different LULCs.

Lastly, we used the CLUE_S model to generate alternative future scenario maps. CLUE_S model is a dynamic, spatially explicit LULC model developed for small regions (e.g., watershed or province). CLUE_S has been widely used throughout the world to develop future LULC scenarios for cities. The model is subdivided into two distinct modules: (1) nonspatial demand, which calculates the area change for all LULC types at an aggregate level and (2) spatially explicit allocation procedure to translate LULC changes to different locations within a study region using a raster-based system. Model conditions must be defined by the user for four categories, representing each scenario: (1) spatial policies and restrictions; (2) land use type specific conversion settings; (3) land use requirements (demand); and (4) location characteristics. We ran CLUE_S using the following inputs: (1) original land use map for 2014 as the reference map; (2) predicted LULC areas for alternative scenarios from the Markov model; and (3) logistic regression model results. We used the Kappa coefficient to evaluate the accuracy of CLUE_S model results, and obtained a Kappa coefficient of 0.92, indicating suitable spatial mapping results.

### Evaluating effectiveness of ERP

We compared four different development scenarios using three criteria: (1) ecosystem composition, (2) ecosystem configuration, and (3) ES. ES were estimated using the above methods for the land use scenarios. To assess ecosystem composition we used ArcGIS 10.0 to measure the percent area and the total area of each land cover type. We analyzed the configuration using FRAGSTATS to calculate: (1) landscape CI and (2) landscape FI. We conducted a paired t-test comparing mean ES levels at the sub-district level (*N* = 236 subdistricts) to determine statistically significant ES tradeoffs among scenarios. We also conducted a correlation analysis on ES for each scenario to evaluate the strength of the synergies among ES.

### Data availability

All relevant data are available upon request from the authors.

## Electronic supplementary material

SUPPLEMENTARY INFO
