# Supplementary material for: Developing China’s Ecological Redline Policy using ecosystem services assessments for land use planning
Source: Nat Commun. 2018 Aug 2;9:3034. doi: 10.1038/s41467-018-05306-1 (PMC6072749; doi:10.1038/s41467-018-05306-1)
Supplement: Supplementary file 1 — SUPPLEMENTARY INFO [file 41467_2018_5306_MOESM1_ESM.pdf]

Developing China's Ecological Redline Policy using ecosystem services assessments for land use planning

Bai et al.

Supplementary Figures

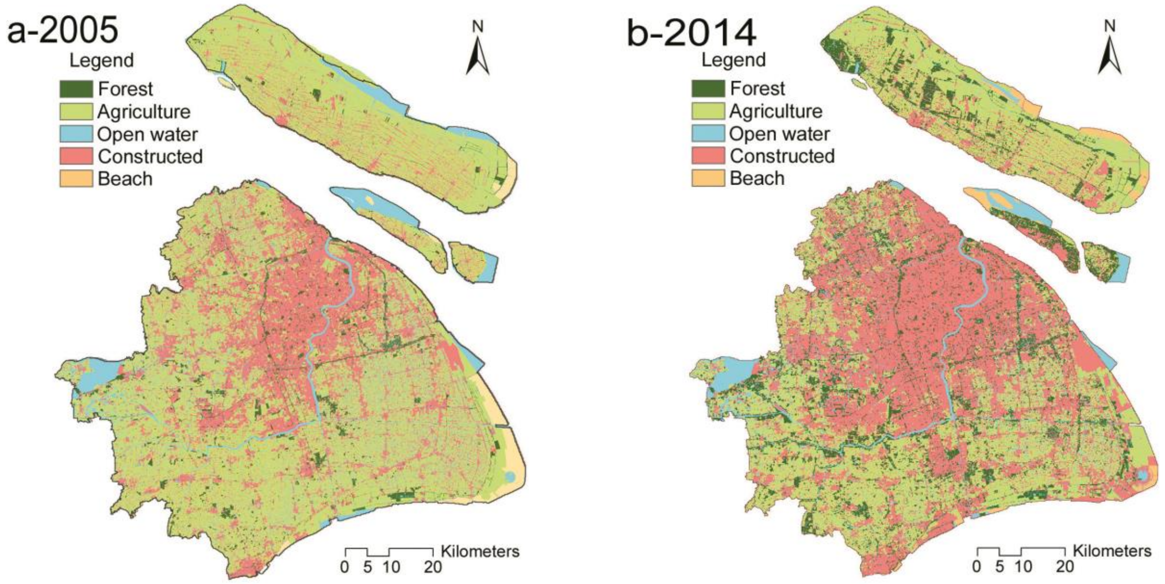

Supplementary Figure 1. Land-use and land-cover maps for (a) 2005 and (b) 2014 for Shanghai Municipality. The maps in this figure were made by the author in ArcGIS software for use in this paper.

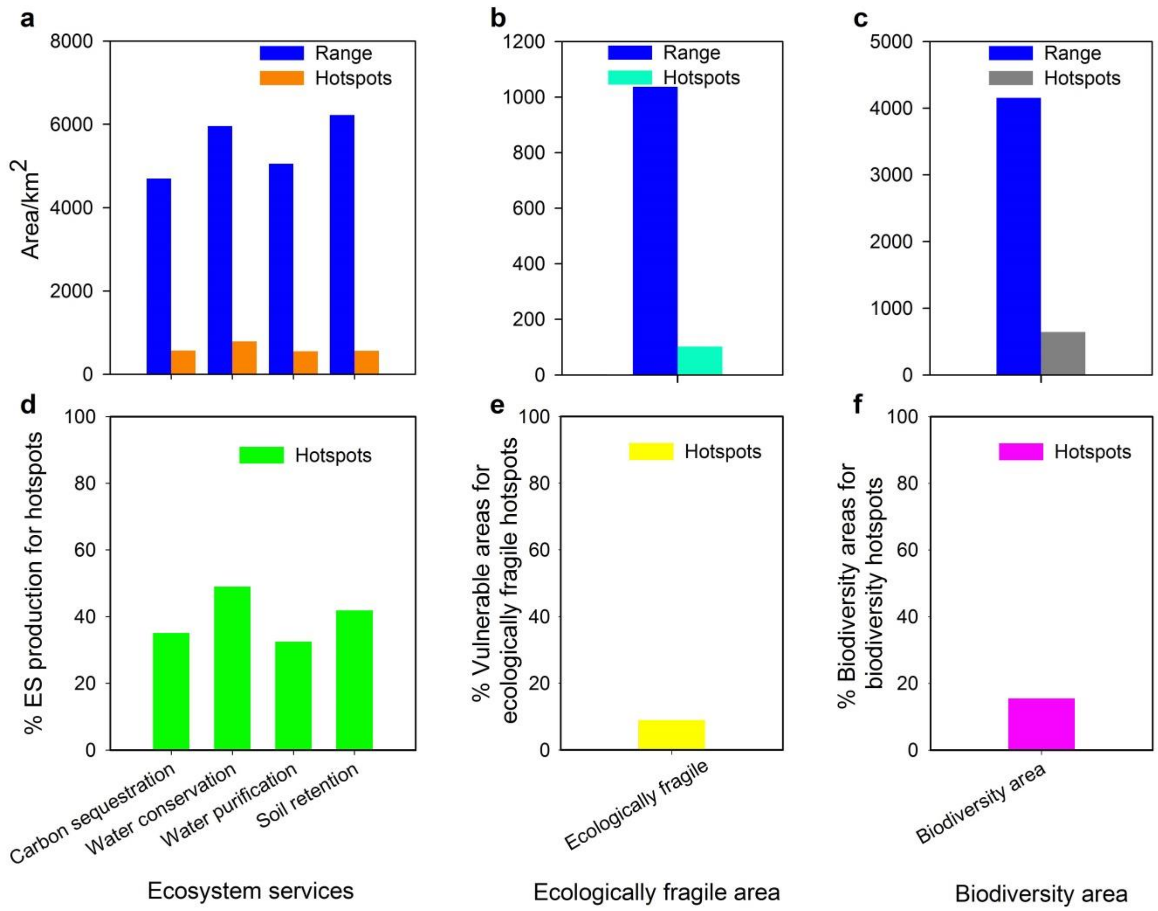

Supplementary Figure 2. **Evaluation of hotspot indicators**. We use a 10% threshold for: ecosystem services, ecologically fragile areas, and biodiversity (a) Total area producing full range of ecosystem services (shown in blue) relative to total area of ecosystem services hotspots (shown in green). (b) Total area of vulnerable areas (shown in blue) relative to total area of ecologically fragile hotspots (shown in yellow). (c) Total area of biodiversity habitat (shown in blue) relative to total area of biodiversity hotspots (shown in magenta). (d) Percent production of ecosystem service hotspots. (e) Percent of vulnerable areas represented by ecologically fragile areas. (f) Percent of suitable habitat for biodiversity represented by biodiversity hotspots. Different colors represent different indicators where green is ecosystem services hotspots, yellow is ecologically fragile hotspots, and magenta is biodiversity hotspots.

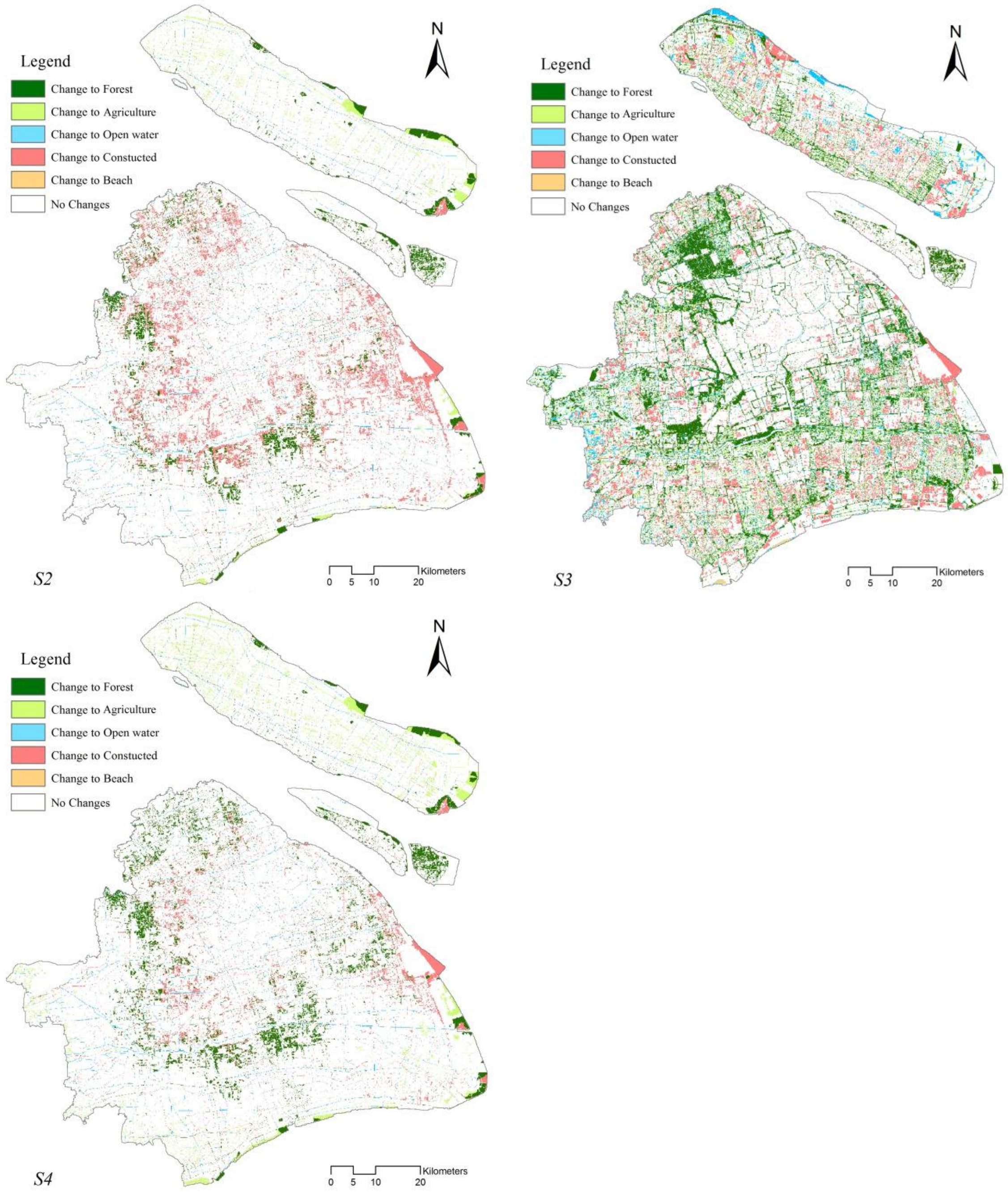

Supplementary Figure 3. **Spatial landscape changes.** Alternative scenarios compared to the baseline scenario (i.e., S1). The maps in this figure were made by the author in ArcGIS software for use in this paper.

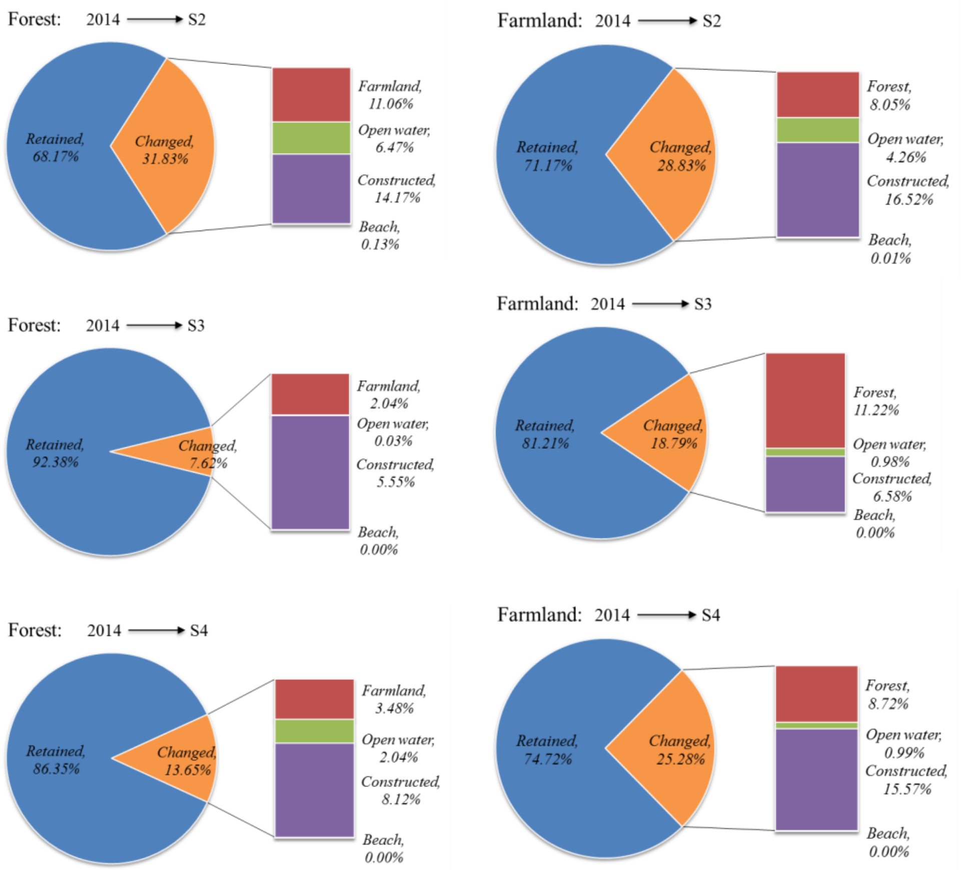

Supplementary Figure 4. Forest-agriculture transition. Influence of alternative scenarios on the forest-agriculture transition compared to the baseline scenario (S1).

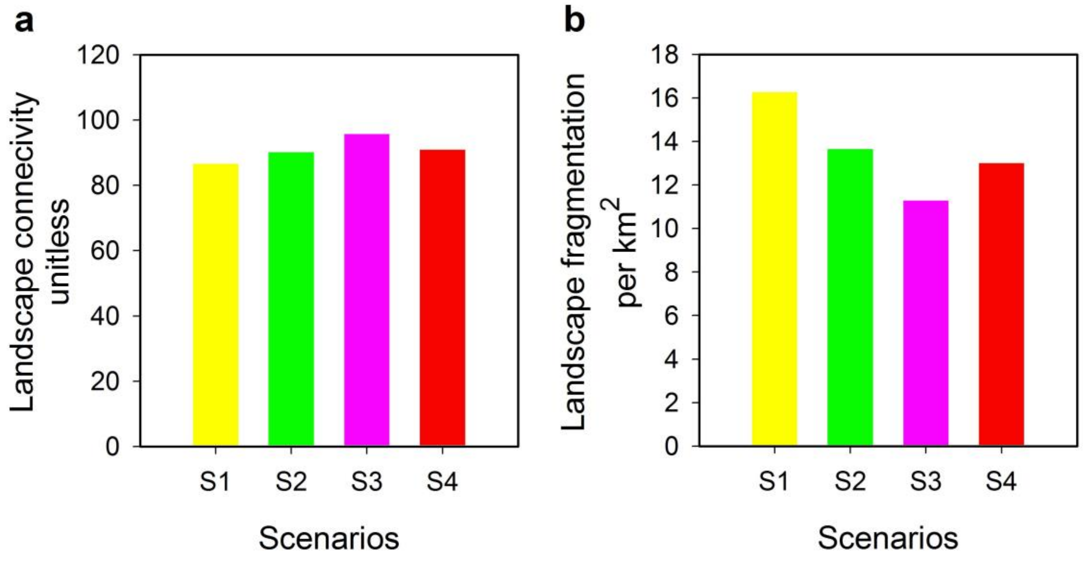

Supplementary Figure 5. Landscape connectivity (a) and fragmentation (b) values for each scenario The different colors represent the different scenarios where yellow is scenario 1, green is scenario 2, magenta is scenario 3 and red is scenario 4.

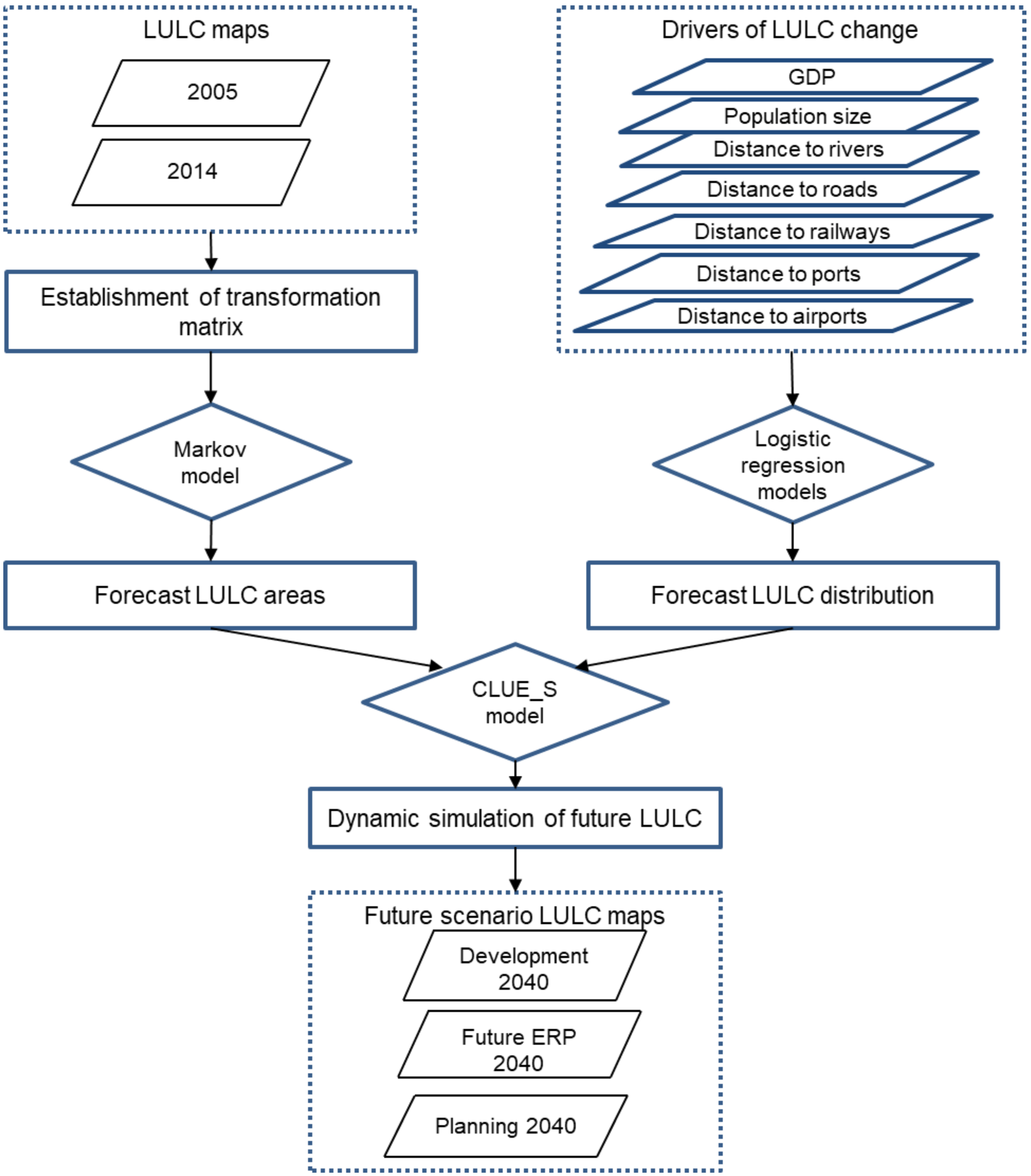

Supplementary Figure 6. Land-use and land-cover (LULC) analysis for projecting alternative future scenarios in Shanghai Municipality for 2040 The Markov model was used to forecast the areas of different LULC types, and logistic regression models were used to forecast LULC spatial distributions. The Conversion and Land Use and its Effects at Small regional extent model (CLUE_S) was used to simulate future LULC to generate the LULC maps for each scenario.

Supplementary Tables

Supplementary Table 1. LULC conversion matrix from 2005-2014, which was used to parameterize the Markov model (km^2)

		2014					
		Forest	Agriculture	Open water	Constructed	Beach	Sum
2005	Forest	304.91	63.88	10.24	60.72	0.08	439.82
	Agriculture	412.60	2130.80	60.03	556.07	0.12	3159.61
	Open water	19.88	59.33	423.67	69.68	0.46	573.02
	Constructed	128.38	136.52	37.98	2185.08	0.65	2488.62
	Beach	2.50	72.26	79.22	44.36	99.63	297.97
	SUM	868.27	2462.79	611.13	2915.91	100.95	6959.05

Supplementary Table 2. Stakeholder groups involved in the spatial planning process

**Category**	**Stakeholder groups**
National government	Ministry of Environmental Protection
Shanghai municipal government	Shanghai Environmental Protection Bureau,<br>Shanghai Municipal Development and Reform Commission, and Shanghai Planning and Land Resources Administration, etc.
Shanghai district and township governments	Districts: Minhang, Baoshan, Jiading, Pudong, Jinshan, Songjiang, Qingpu, Fengxian, and Chongming.
Scientific institutions	Shanghai Academy of Environmental Sciences, Shanghai Urban Planning and Design Research Institute, Shanghai Planning and Land Resources Administartion, Fudan University, Tongji University, Shanghai Municipal Institute of Surveying and Mapping, and Shanghai Ocean Planning and Design Research Institute, etc.
Agriculture	Local farmers
Public	Urban residents

Supplementary Table 3. Ecosystem composition of each scenario as percent area (%) and total area (km^2) for each land-cover type

Ecosystems	S1/2014		S2/2040		S3/2040		S4/2040	
	Area	Percentage	Area	Percentage	Area	Percentage	Area	Percentage
**Forest**	868.27	12.48%	1074.09	15.43%	1455.35	20.91%	1160.49	16.68%
**Agriculture**	2462.79	35.39%	1915.76	27.53%	2177.84	31.30%	2159.75	31.04%
**Open water**	611.13	8.78%	595.11	8.55%	612.56	8.80%	593.49	8.53%
**Constructed**	2915.91	41.90%	3343.87	48.05%	2614.21	37.57%	3010.1	43.25%
**Beach**	100.95	1.45%	30.22	0.43%	99.08	1.42%	35.22	0.51%
**Total:**	6959.05	100%	6959.05	100%	6959.05	100%	6959.05	100%

Supplementary Table 4. Correlation among four ecosystem services for each scenario. **denote significant correlation at significant level of 0.01

Scenarios	Block samples	Ecosystem services		
	n=236	Carbon Sequestration	Water Conservation	Water Purification
S1	Water Conservation	0.923**		
	Water Purification	0.815**	0.939**	
	Soil Retention	0.634**	0.776**	0.805**
S2	Water Conservation	0.928**		
	Water Purification	0.796**	0.912**	
	Soil Retention	0.586**	0.747**	0.809**
S3	Water Conservation	0.963**		
	Water Purification	0.877**	0.955**	
	Soil Retention	0.660**	0.763**	0.810**
S4	Water Conservation	0.923**		
	Water Purification	0.795**	0.927**	
	Soil Retention	0.580**	0.747**	0.809**

Supplementary Table 5. Ministry of Environmental Protection (2015) criteria for determining ecological redlines.

#	**Step**	**Criteria**
1	Identify ecological important areas	Determine and evaluate: ecosystem services, ecologically fragile areas, and biodiversity conservation.
2	Establish ecological redline areas	Use the information from step 1 to determine ecological redline areas.
3	Establish ecological redline areas database	Compile information on the ERAs: distribution, area, range, and geographical coordinates.

Supplementary Table 6. Accuracy assessment of classified 2014 image

	LULC classes (reference)							User's Accuracy
	Samples	Forest	Agriculture	Open water	Constructed	Beach	Totals	
LULC classes (classified)	Forest	115	3	0	0	1	119	**96.64%**
	Agriculture	4	94	1	5	2	106	**88.68%**
	Open water	0	0	75	0	3	78	**96.15%**
	Constructed	0	4	0	83	0	87	**95.40%**
	Beach	1	3	2	0	72	78	**92.31%**
	Totals	120	104	78	88	78	468	
Producer's Accuracy		**95.83%**	**90.38%**	**96.15%**	**94.32%**	**92.31%**		**93.80%**

Supplementary Table 7. Input values for the InVEST 3.2.0 carbon storage and sequestration model (tonnes/ha)

**Land use/land cover type**	**Aboveground biomass**	**Belowground biomass**	**Soil**	**Dead organic matter**
Residential	0	0	0	0
Commercial	0	0	0	0
Industrial	0	0	0	0
Road	0	0	0	0
River	0	0	0	0
Lake	0	0	0	0
Reservoir	0	0	0	0
Beach	12	6	24	0
Pond	0	0	0	0
Field crop	3	2	8	1
Irrigated paddy field	2	1	10	0
Garden plot	150	30	120	10
Grass	4	4	15	1
Forest	175	120	130	55
Bare land	0	0	0	0

Data source: InVEST library and literature 1, 2 and 3.

Supplementary Table 8. Input values for the InVEST 3.2.0 water yield model

**Land use/land cover type**	**Max root depth (mm)**	**Evapotranspiration coefficient**
Residential	500	0.4
Commercial	500	0.4
Industrial	500	0.4
Road	500	0.4

River	500	1
Lake	500	1
Reservoir	500	1
Beach	3500	0.9
Pond	500	1
Field crop	1800	0.6
Irrigated paddy field	1800	0.6
Garden plot	3000	0.7
Grass	2400	0.6
Forest	6500	1
Bare land	2000	0.6

Supplementary Table 9. Nitrogen export and filtration coefficients for InVEST 3.2.0 nutrient retention model

**Land use/land cover type**	**Nitrogen export coefficients (kg*ha^{-1}*y^{-1})**	**Nitrogen filtration coefficients**
Residential	8.5	0.05
Commercial	14.5	0.05
Industrial	9.5	0.05
Road	13	0.05
River	0.001	0.05
Lake	0.001	0.05
Reservoir	0.001	0.05
Beach	1.5	0.85
Pond	0.001	0.05
Field crop	11.1	0.15
Irrigated paddy field	11.1	0.15
Garden plot	9.5	0.35
Grass	10.5	0.25
Forest	1.8	0.8
Bare land	3.5	0.05

Supplementary Table 10. Input data for InVEST 3.2.0 sediment delivery ratio model

Land use/land cover type	Cover and management factor	Management practice factor	Sediment retention value (%)
Residential	0.001	0.001	0.05
Commercial	0.001	0.001	0.05
Industrial	0.001	0.001	0.05
Road	0.001	0.001	0.05
River	0.001	0.001	0.05
Lake	0.001	0.001	0.05
Reservoir	0.001	0.001	0.05
Beach	0.002	0.001	0.5
Pond	0.001	0.001	0.05
Field crop	0.02	0.012	0.38
Irrigated paddy field	0.02	0.012	0.38
Garden plot	0.006	0.005	0.48
Grass	0.005	0.002	0.49
Forest	0.003	0.002	0.55
Bare land	0.005	0.002	0.05

Supplementary Table 11. Evaluation method using expert opinion to estimate desertification sensitivity

Indicators	Insensitive	Slight sensitivity	Medium sensitivity	High sensitivity	Extreme sensitivity
Humidity index	>0.65	0.5-0.65	0.20-0.50	0.05-0.20	<0.05
Wind speed > 6 m/s (number of days)	<15	15-30	30-45	45-60	>60
Soil texture	Bedrock	Viscidity	Gravel	Loam	Sandiness
Vegetation cover	Dense	Moderate	Less	Sparse	Bare
Expert Grade	1	3	5	7	9

Supplementary Table 12. Evaluation method using expert determine to estimate salinization sensitivity

Indicators	Insensitive	Slight sensitivity	Medium sensitivity	High sensitivity	Extreme sensitivity
Evaporation/Precipitation	<1	1-3	3-10	10-15	>15
Groundwater mineralization	<1	1-5	5-10	10-25	>25
Topography	Mountainous	Diluvial plain, delta	Flood alluvial plain	Valley plain	Low coastal plain, closed stream basin
Grade	1	3	5	7	9

Supplementary Table 13. Sensitivity of different habitat types to each anthropogenic stressor

Land use/land cover type	Habitat	Residential	Commercial	Industrial	Road	Field crop	Irrigated paddy field
Residential	0	0	0	0	0	0	0
Commercial	0	0	0	0	0	0	0
Industrial	0	0	0	0	0	0	0
Road	0	0	0	0	0	0	0
River	0.5	0.9	0.9	0.9	0.8	0.7	0.7
Lake	1	0.9	0.9	0.9	0.8	0.7	0.7
Reservoir	0.8	0.9	0.9	0.9	0.8	0.7	0.7
Beach	1	0.9	0.9	0.9	0.8	0.7	0.7
Pond	0	0	0	0	0	0	0
Field crop	0.3	0.5	0.5	0.5	0.3	0.3	0.3
Irrigated paddy field	0.3	0.5	0.5	0.5	0.3	0.3	0.3
Garden plot	1	0.5	0.5	0.5	0.3	0.3	0.3
Grass	0.5	0.6	0.6	0.6	0.4	0.4	0.4
Forest	1	0.8	0.8	0.8	0.7	0.6	0.6
Bare land	0	0	0	0	0	0	0

Supplementary Table 14. Relative impact of each stressor on different habitats

Stressor	Max Distance[a]	Weight[b]	Decay[c]
Residential	10	1	0
Commercial	12	1	0
Industrial	12	1	0
Road	2	0.8	0
Field crop	6	0.7	0
Irrigated paddy field	6	0.7	0

Note: [a] Distance over which each stressor affects biodiversity (km); [b] Impact of each stressor on biodiversity relative to other stressors by indicating whether the impact of the stressor decreases linearly, more steeply or exponentially with distance from the source of the threat; [c] Value is 0 or 1 where a value of 1 indicates a linear decline in impact, while 0 indicates an exponential decline.

Supplementary Table 15. LULC transition probability matrix, 2005-2014

Transfer from		2014				
	Types	Forest	Agriculture	Open water	Constructed	Beach
2005	Forest	35.12%	2.59%	1.68%	2.08%	0.08%
	Agriculture	47.52%	86.52%	9.82%	19.07%	0.12%
	Open water	2.29%	2.41%	69.33%	2.39%	0.46%
	Constructed	14.79%	5.54%	6.21%	74.94%	0.64%
	Beach	0.29%	2.93%	12.96%	1.52%	98.69%
Transfer to		2014				
	Types	Forest	Agriculture	Open water	Constructed	Beach
2005	Forest	69.33%	14.52%	2.33%	13.81%	0.02%
	Agriculture	13.06%	67.44%	1.90%	17.60%	0.00%
	Open water	3.47%	10.35%	73.94%	12.16%	0.08%
	Constructed	5.16%	5.49%	1.53%	87.80%	0.03%
	Beach	0.84%	24.25%	26.59%	14.89%	33.44%

Supplementary Table 16. Beta values[1] for regression results of the spatial distribution of LULC in Shanghai

Driver	Type of ecosystem				
	Constructed	Forest	Agriculture	Open water	Beach
Constant	1.182085	-2.055922	-1.542633	-2.988958	-11.744971
Distance to harbor	-0.007867	-0.002328	0.001921	0.016908	-0.034646
Distance to airport	-0.010904	0.007446	0.007492	-0.001068	0.046976
Distance to city-level river	0.001568	-0.002021	-0.00013	0.002275	0.006314
Distance to district-level river	-0.009291	0.015929	-0.007718	0.015733	0.0454
Distance to town/village-level river	0.034594	-0.017309	-0.066787	-0.000464	0.023346
Distance to surface road	-0.053264	-0.012115	0.025026	0.016102	0.029956
Distance to metro	-0.007801	-0.003258	0.010929	-0.004143	0.039339
Distance to railway	-0.002367	0.000416	0.001132	0.000168	0.002121
ROC value	0.9232	0.9134	0.8825	0.9028	0.9103

[1] All variables significant at $p < 0.01$.

Supplementary Methods

Human benefits from selected ecosystem services. Shanghai is located on an alluvial plain at the outlet of the Yangtze River, and is believed to be the most vulnerable city in China to climate change[4]. Shanghai is a low-lying city that is highly vulnerable to climate change, such as rising sea levels, extreme weather, drier wet seasons, and increased summer temperatures[5]. Climate change threatens Shanghai's economy in many ways: (1) declining fisheries from rising sea temperatures; (2) typhoons and other extreme weather events cause local damages and threaten tourism; (3) coastal erosion from sea level rise is washing away precious delta soil thereby threatening infrastructure and water supplies[5]. China is committed to reducing its carbon emissions through energy efficiency, renewable energy, and climate change mitigation actions like carbon sequestration. Shanghai aims to reduce its peak carbon emissions by 15% by 2020 to help meet the national carbon emission reduction target. To reduce the negative social impacts of climate change – the Shanghai Government wants to invest in ecosystems to enhance local carbon sequestration. As the largest, most modernized city in China, Shanghai has experienced extensive urbanization, which has dramatically altered its ecosystems. Shanghai now only has small patches of natural secondary forests in Sheshan Hill and Jinshan Island. Shanghai's urban forest is comprised of roughly 113 tree species, 85 shrubs, and 534 grass taxa[6]. Scientists estimate Shanghai's forest biomass carbon is 0.48 Tg in the urban area, and the forest soil organic carbon is 2.48 Tg in urban and suburban areas. This is considered a low carbon sequestration level relative to other Chinese megacities[7]. Hence the Shanghai Government wants the ecological redline areas to increase local carbon sequestration for the human benefit of contributing to the mitigation of global climate change impacts.

Water scarcity in terms of sustaining water supplies and improving water quality are important human benefits for people who live in Shanghai. Shanghai has access to sufficient water due to its proximity to the Yangtze River. Scholars, however, have conducted a multitude of studies indicating concern about future freshwater supplies due to rapid increases in water demand and poor water management practices[8]. Since 2010 the major freshwater source for Shanghai has been the Qingcaosha Reservoir near the mouth of the Yangtze River, which supplies over 50% of Shanghai's freshwater. Hence stakeholders want the human benefit of continuous water supply from freshwater sources (e.g., Qingcaosha Reservoir and Huangpu River) in Shanghai, therefore they selected water conservation. The major problem causing water scarcity in Shanghai is water pollution leading to poor water quality across Shanghai. In 2015, Shanghai's Environment Monitoring Center tested 100 spots across the city and found only 10% of the waterways were either "excellent" or "good water quality". Over 65% of the freshwater in Shanghai was graded as heavily polluted. Therefore stakeholders are highly concerned about water quality and want to invest in ecosystems to improve water quality, specifically for human benefits of drinking water, recreation, and fisheries. Hence we selected water purification.

Lastly, in Shanghai the land degradation causing the loss of fertile lands is soil erosion. In China soil erosion is a national dilemma since it is estimated that almost 40 percent of China's territory (over 3.5 million km^2) suffers from soil erosion. Soil erosion can be found in almost every river basin and every province in China. Shanghai has a lower soil erosion rate than other portions of China; however the Yangtze River Basin has the largest soil erosion area (approximately 28% of the national total)[9]. Given the national significance as well as high importance of soil erosion in the Yangtze River Basin, stakeholders selected soil erosion control

for the human benefits of: fertile soils for agriculture, mitigation of lake/reservoir siltation, food security from soil contamination, and flood control.

Land use and land cover (LULC) analysis. Multi-temporal aerial images (spatial resolution 0.5 m) were used to extract LULC information for 2005 and 2014. We obtained 41 images from the Shanghai Urban Development Information Research Center who used helicopters to obtain aerial images from January-March, 2005 and January-March, 2014. We adjusted original aerial images using the Universal Transverse Mercator (UTM) projection system, and we merged the images together in ERDAS Imagine 9.3. (Norcross GA: Hexagon Geospatial, Inc). LULC information was extracted using manual visual interpretation in ArcGIS 10.0 (Redlands, CA: ESRI, Inc). We delineated the LULC creating polygons for the five main categories: (1) forest; (2) agriculture; (3) constructed land; (4) open water; (5) beach. For instance, polygons for rivers and roads were created then delineated other LULC polygons by clipping existing polygons or adding new polygons. The five main categories were further divided into 15 sub-categories, namely residential land, commercial land, industrial land, road, river, lake, reservoir, beach, pond, field crop, irrigated paddy field, garden plot, grass, forest, and bare land. Uncertainty of LULC classifications are commonly expressed as accuracy reports via error matrices. Error matrices compare on a class-by-class basis, the relationship between reference data and the corresponding results of the software classification. We used field GPS points to generate the reference data. We calculated the total accuracy, user's accuracy, and producer's accuracy for the 2014 LULC classification. Total accuracy of the classification for 2014 with ground-based survey data was 93.80% (Supplementary Table 6). To facilitate the following analyses, the LULC layers were converted to a grid format at a spatial resolution of 50 m.

Assessment indicators. We use three main indicators to standardize the Ministry of Environmental Protection (MEP) official criteria for determining the ERAs: (1) ES hotspots, (2) ecologically fragile hotspots, and (3) biodiversity hotspots. Also we use three additional indicators to assess ERAs: (1) ecosystem composition, (2) landscape fragmentation, and (3) landscape connectivity.

Norman Myers developed the term "hotspots" in the 1980s to identify areas of high species richness, endemism and/or threat, which has widely been used to prioritize areas for biodiversity conservation. We extend the "hotspot" concept to ES and ecologically fragile areas. Studies have suggested that the spatial variation in biodiversity and other ES are not necessarily positively correlated (highly dependent on the ES type)[10], thus creating distinct criteria for each hotspot can help managers prioritize for these three important (sometimes distinct) aspects of ecosystems. Several studies have used percentages (top 10% or top 20%) of ES production to determine thresholds for ES hotspots to formulate a systematic methodology while also producing feasible areas for protection[11,12]. In South Africa, Egoh et al.[13] examined carbon sequestration, water resources conservation, and soil conservation. They found most of ES production occurred in ecosystem areas representing only 5-10% of South Africa's land area. Ecologically fragile hotspots are areas that have been highly degraded from stressors, making them very sensitive to "tipping points" where the ecosystem state flips from "desirable state" to an alternative state that is less beneficial to human welfare and biodiversity[14]. In China, major stressors tied to ecosystem management (excluding pollutants and urbanization) causing large-scale alterations in ecosystem functionality are: soil erosion, desertification, and salinization[15]. Biodiversity hotspots have been widely used to help managers identify high concentrations of species richness and endemism that are undergoing high habitat loss. It provides a useful way to

identify key ecosystem areas that must be protected to sustain as much biodiversity as possible given financial and space limitations due to competing interests. Myers et al.[16] found that 44% of all species of vascular plants and 35% of all species in four vertebrate groups reside in 25 hotspots, comprising only 1.4% of the Earth's land surface. Incorporation of the different types of hotspots into spatial planning can possibly help policymakers craft more comprehensive strategies for improving how cities balance environmental protection and development.

Scientists have shown that ES depends on biodiversity and ecosystem functions, which are impacted by alterations in landscape structure: composition and configuration. Ecosystem composition is defined as the amount of each land use and land cover (LULC) type. Ecosystem configuration is the spatial arrangement of LULC types. Lamy et al.[17] found that the distribution of ES on the landscape depends on both the overall composition of LULC types and their configuration on the landscape. We measure ecosystem composition as (1) percent area of each land-cover type and (2) total area of each landscape type. We measure ecosystem configuration as (1) landscape connectivity using a connectivity index and (2) landscape fragmentation using a fragmentation index. Landscape connectivity is an index to describe the degree to which the landscape facilitates or impedes movement among resource patches. Landscape connectivity is a function of the percentage of a landscape occupied by that habitat type, and plays a vital role in protecting biodiversity, and maintaining ecosystem stability and integrity[18]. In comparison, landscape fragmentation is an index to describe the degree to which a habitat or LULC type is broken into smaller, disconnected parcels. It reflects pattern changes caused by natural factors or human activities[19]. Inclusion of landscape connectivity and land fragmentation as explicit components of landscape structure are useful for comparing ERP to other land-use scenarios.

Social survey. No permits were needed to conduct our surveys with human subjects since our surveys were used for non-commercial purposes. Prior to surveying individuals, we provided each person with a clear statement explaining the purpose of the survey and the type of personal information to be collected. We clearly articulated that the personal information would only be used for scientific research, and any personal information would remain confidential. Also we clearly explained that any results from the surveys would be presented in aggregate with no personal identifying information. After clarifying the purpose of the research and the privacy statement we then asked each individual if they would be willing to participate in the survey.

The gender make-up of the surveyed population is: (1) 44% is females; (2) 56% is males. The age make-up of the surveyed population is: (1) 28% are under 30; (2) 34% are 31-40; (3) 20% are 41-50; (4) 10% are 51-60; (5) 8% are older than 60. The professional backgrounds are classified as: (1) government staff (11%); (2) teachers and researchers (28%); (3) company employees (24%); (4) industrial enterprise (20%); (5) professional staff, such as lawyers and doctors (8%); (6) other professions (3%); (7) full-time students (6%). We also analyzed people's ES preferences in terms of residence. We found that people who lived in the city center were more likely to rank water purification as a priority ES because water quality problems are worse in the city center compared to the suburban and exurban areas. Also people who lived in exurban areas, especially in coastal zones were more aware of the importance of soil control due to their proximity to the coast giving them increased awareness of coastal erosion.

Stakeholder engagement. The Shanghai Municipal Government (SMG), Shanghai Municipal Planning and Land Resources Administration (SPLRA) and Shanghai Environmental Protection

Bureau (SEPB) held two types of consultations to facilitate the selection of the targeted ERAs for Shanghai.

A. District-level: The purpose is to obtain agreement on the location of ERAs at the district-level. First the SPLRA invited leaders of district governments, and scientists with ecosystem/natural resources and spatial planning expertise. In summary, the main stakeholders for the district-level workshops were: (1) district government leaders, mainly from suburban districts where the majority of the ERAs are located; (2) interdisciplinary team of scientific experts spanning institutions (i.e., Shanghai Academy of Environmental Sciences, Shanghai Urban Planning and Design Research Institute, Fudan University, Tongji University, Shanghai Municipal Institute of Surveying and Mapping, and Shanghai Ocean Planning and Design Research Institute). During the scoping workshops, spatial maps were first presented to district governments and scientists using powerpoint presentations. They also provided all stakeholders with the data. Stakeholders discussed the spatial distribution of the ERAs, which entailed us educating stakeholders on the scientific criteria, and explaining the analysis. Answering questions on the scientific analysis helped build stakeholder confidence on the credibility of the assessment, and for scientists the scoping workshops helped legitimize the scientific analysis.

B. Public comment. After the district-level consultations, the revised ERAs were posted online to the public. Citizens could freely download this document and provide public comment to help refine the targeted ERAs.

Disagreements stemmed from stakeholders wanting certain areas to be available for future development. District governments outlined the areas of disagreement to the SEPB who then provided the proposed ERA adjustments to the scientists. The scientists examined how the proposed ERA reductions may impact ES, ecologically fragile areas, and biodiversity. We

subsequently provided this information on the potential losses to the SEPB. The SEPB weighed any losses on the three ecological criteria relative to stakeholder concerns, and modified the maps accordingly. They sent the modified maps back to the district governments. Finally the SEPB and the district governments had to compromise on certain areas. They built consensus by offering future compensation to impacted stakeholders. Local communities eventually agreed to the majority of ERAs because of its legal importance. Not following the ERAs could be quite risky in terms of higher taxes and future penalties. The Ecological Redline Policy is incredibly important in China, thus local governments know they must strive for strict protection of the identified ecological areas.

Supplementary References

1. Zhang, J.F. *et al.* Investigation on distribution and biomass of Halophytes at beach in Shanghai. *Acta. Agric. Jiangxi* **27**, 26-29 (2015).
2. Li, G.Y. *et al.* Variation of spatial estimation and distribution of vegetation biomass in Yangtze river delta during 2000-2010. *Journal of Ecology and Rural Environment* **32**, 708-715 (2016).
3. Intergovernmental Panel on Climate Change (IPCC). *2006 IPCC Guidelines for National Greenhouse Gas Inventories*. Institute for Global Environmental Strategies, Japan (2006).
4. Gong, Y.D. and Yu, F. Shanghai Tackles Climate Change. *Shanghai Daily*. December **21**, (2009). https://www.shine.cn/archive/feature/Shanghai-tackles-climate-change/.
5. Liu, C. and Wire, C. Shanghai struggles to save itself from the sea. Scientific American. Semptember **27**, (2011). https://www.scientificamerican.com/article/shanghai-struggles-to-save-itself-from-east-china-sea/
6. Yao, X. *et al*. What Causal Drivers Influence Carbon Storage in Shanghai, China's Urban and Peri-Urban Forests? *Sustainability,* **9**, 577 (2017).
7. Wang, Z. *et al*. Characteristics of carbon storage in Shanghai's urban forest. *Chin Sci Bull*. **58**, 1130-1138 (2013).
8. Li, M.T. *et al*. Estimating urban water demand under conditions of rapid growth: the case of Shanghai. *Reg Environ Change*. **17**,1153-1161(2017).
9. Rao, E.M. *et al*. National assessment of soil erosion and its spatial patterns in china, *Ecosyst. Hea. Sustain*. **1**, 1-10 (2015).
10. Ricketts, T.H. *et al.* Disaggregating the evidence linking biodiversity and ecosystem services. *Nat. Commun.* **7**, 13106 (2016).
11. Qiu, J. and M.G. Turner. Spatial interactions among ecosystem services in an urbanizing agricultural watershed. *Proc. Natl. Acad. Sci. USA* **110**, 12149-12154 (2013).

12. Eigenbrod, F. *et al.* The impact of proxy-based methods on mapping the distribution of ecosystem services. *J. Appl. Ecol.* **47**, 377-385 (2010).
13. Egoh, B. *et al.* Mapping ecosystem services for planning and management. *Agric. Ecosyst. Environ.* **127**, 135-140 (2008).
14. Chen, L. *et al.* Towards sustainable integrated watershed ecosystem management: a case study in Dingxi on the Loess Plateau, China. *Environ. Manage.* **51**, 126-137 (2013).
15. People's Republic of China Ministry of Environmental Protection (MEP). *Technical guide for ecological red line*. No. 2015.5. Chinese Ministry of Environmental Protection, Beijing, China (2015).
16. Myers, N. *et al.* Biodiversity hotspots for conservation priorities. *Nature*, **403**, 853-858 (2000).
17. Lamy, T. *et al.* Landscape structure affects the provision of multiple ecosystem services. *Environ. Res. Lett.* **11**, 1-9 (2016).
18. Taylor. P.D. *et al.* Connectivity is a vital element of landscape structure. *Oikos* **68**, 571-573 (1993).
19. Hulshoff, R.M. Landscape indices describing a Dutch landscape. *Landscape Ecol.* **10**, 101-111 (1995).
